# Supplementary material for: Scalable, high quality, whole genome sequencing from archived, newborn, dried blood spots
Source: NPJ Genom Med. 2023 Feb 14;8:5. doi: 10.1038/s41525-023-00349-w (PMC9929090; doi:10.1038/s41525-023-00349-w)

**Supplementary Material**

**Supplementary Tables**

**Supplementary Table 1. Sample characteristics and number of DBS made for each sample.** Abbreviations: FTA: FTA card; PC: protein saver card; KAPA: KAPA Hyper Plus library method; Qiagen: Qiagen lysis method for DNA isolation; Illumina: Tagmentation PCR-free library method; #: the spot from CDPH was double size of simulated DBS.

| **Sample ID** | **Simulated DBS or CDPH DBS?** | **Number of simulated DBS made** | **Number of DBS punches used for Isolation** | **Age of DBS at gDNA isolation** | **DBS paper type** | **DNA extraction method** | **Library preparation method** | **DNA used for library preparation (ng)** | **DRAGEN version** |
| --- | --- | --- | --- | --- | --- | --- | --- | --- | --- |
| Subject 1 | Simulated DBS | 8 | 6 | 52 | FTA | Illumina | Illumina | 267.60 | 2.1.5 |
| Subject 1 | Simulated DBS |  | 10 | 58 | FTA | Qiagen | KHP | 420.90 | 2.1.5 |
| Subject 1 | Simulated DBS | 10 | 6 | 52 | PC | Illumina | Illumina | 246.60 | 2.1.5 |
| Subject 1 | Simulated DBS |  | 10 | 8 | PC | Qiagen | KHP | 300.00 | 2.1.5 |
| Subject 1 | Simulated DBS |  | 10 | 37 | PC | Qiagen | KHP | 526.65 | 2.1.5 |
| Subject 10 | Simulated DBS | 2 | 6 | 52 | FTA | Illumina | Illumina | 169.20 | 2.1.5 |
| Subject 10 | Simulated DBS |  | 10 | 58 | FTA | Qiagen | KHP | 481.50 | 2.1.5 |
| Subject 10 | Simulated DBS | 3 | 6 | 52 | PC | Illumina | Illumina | 210.60 | 2.1.5 |
| Subject 10 | Simulated DBS |  | 10 | 8 | PC | Qiagen | KHP | 300.00 | 2.1.5 |
| Subject 10 | Simulated DBS |  | 10 | 58 | PC | Qiagen | KHP | 339.18 | 2.1.5 |
| Subject 11 | Simulated DBS | 8 | 6 | 52 | FTA | Illumina | Illumina | 500.00 | 2.1.5 |
| Subject 11 | Simulated DBS |  | 6 | 64 | FTA | Illumina | Illumina | 283.20 | 2.1.5 |
| Subject 11 | Simulated DBS |  | 10 | 183 | FTA | Qiagen | KHP | 522.80 | 2.1.5 |
| Subject 11 | Simulated DBS | 10 | 6 | 53 | PC | Illumina | Illumina | 500.00 | 2.1.5 |
| Subject 11 | Simulated DBS |  | 10 | 9 | PC | Qiagen | KHP | 300.00 | 2.1.5 |
| Subject 11 | Simulated DBS |  | 10 | 38 | PC | Qiagen | KHP | 380.98 | 2.1.5 |
| Subject 12 | Simulated DBS | 4 | 10 | 37 | FTA | Qiagen | KHP | 571.17 | 2.1.5 |
| Subject 12 | Simulated DBS | 2 | 10 | 8 | PC | Qiagen | KHP | 300.00 | 2.1.5 |
| Subject 12 | Simulated DBS |  | 10 | 37 | PC | Qiagen | KHP | 357.46 | 2.1.5 |
| Subject 13 | Simulated DBS | 4 | 6 | 52 | FTA | Illumina | Illumina | 500.00 | 2.1.5 |
| Subject 13 | Simulated DBS |  | 6 | 64 | FTA | Illumina | Illumina | 501.30 | 2.1.5 |
| Subject 13 | Simulated DBS | 2 | 6 | 37 | PC | Illumina | Illumina | 500.00 | 2.1.5 |
| Subject 13 | Simulated DBS |  | 10 | 9 | PC | Qiagen | KHP | 300.00 | 2.1.5 |
| Subject 14 | Simulated DBS | 4 | 6 | 52 | FTA | Illumina | Illumina | 206.70 | 2.1.5 |
| Subject 14 | Simulated DBS | 2 | 6 | 52 | PC | Illumina | Illumina | 186.90 | 2.1.5 |
| Subject 14 | Simulated DBS |  | 10 | 8 | PC | Qiagen | KHP | 300.00 | 2.1.5 |
| Subject 15 | Simulated DBS | 2 | 10 | 74 | FTA | Qiagen | KHP | 300.00 | 2.1.5 |
| Subject 15 | Simulated DBS | 2 | 10 | 74 | PC | Qiagen | KHP | 300.00 | 2.1.5 |
| Subject 16 | Simulated DBS | 2 | 10 | 99 | PC | Qiagen | KHP | 276.63 | 2.1.5 |
| Subject 17 | Simulated DBS | 2 | 10 | 77 | PC | Qiagen | KHP | 279.75 | 2.1.5 |
| Subject 18 | Simulated DBS | 2 | 6 | 6 | FTA | Illumina | Illumina | 245.70 | 2.1.5 |
| Subject 18 | Simulated DBS | 2 | 6 | 6 | PC | Illumina | Illumina | 301.80 | 2.1.5 |
| Subject 19 | Simulated DBS | 2 | 6 | 6 | FTA | Illumina | Illumina | 171.90 | 2.1.5 |
| Subject 19 | Simulated DBS | 2 | 6 | 6 | PC | Illumina | Illumina | 177.60 | 2.1.5 |
| Subject 2 | Simulated DBS | 2 | 10 | 58 | FTA | Qiagen | KHP | 1003.80 | 2.1.5 |
| Subject 2 | Simulated DBS | 2 | 10 | 8 | PC | Qiagen | KHP | 300.00 | 2.1.5 |
| Subject 2 | Simulated DBS |  | 10 | 58 | PC | Qiagen | KHP | 852.90 | 2.1.5 |
| Subject 20 | Simulated DBS | 2 | 6 | 439 | FTA | Illumina | Illumina | 178.50 | 2.1.5 |
| Subject 21 | Simulated DBS | 2 | 6 | 397 | FTA | Illumina | Illumina | 228.00 | 2.1.5 |
| Subject 22 | Simulated DBS | 2 | 6 | 380 | FTA | Illumina | Illumina | 214.50 | 2.1.5 |
| Subject 23 | Simulated DBS | 2 | 6 | 636 | FTA | Illumina | Illumina | 154.80 | 2.1.5 |
| Subject 24 | Simulated DBS | 2 | 6 | 661 | FTA | Illumina | Illumina | 191.10 | 2.1.5 |
| Subject 25 | Simulated DBS | 2 | 6 | 122 | FTA | Illumina | Illumina | 199.20 | 2.1.5 |
| Subject 3 | Simulated DBS | 2 | 10 | 122 | FTA | Qiagen | KHP | 738.30 | 2.1.5 |
| Subject 3 | Simulated DBS | 2 | 10 | 87 | PC | Qiagen | KHP | 300.00 | 2.1.5 |
| Subject 3 | Simulated DBS |  | 10 | 101 | PC | Qiagen | KHP | 369.62 | 2.1.5 |
| Subject 4 | Simulated DBS | 4 | 6 | 104 | FTA | Illumina | Illumina | 312.00 | 2.1.5 |
| Subject 4 | Simulated DBS |  | 10 | 110 | FTA | Qiagen | KHP | 587.40 | 2.1.5 |
| Subject 4 | Simulated DBS | 2 | 6 | 104 | PC | Illumina | Illumina | 417.00 | 2.1.5 |
| Subject 4 | Simulated DBS |  | 10 | 75 | PC | Qiagen | KHP | 300.00 | 2.1.5 |
| Subject 5 | Simulated DBS | 2 | 10 | 110 | FTA | Qiagen | KHP | 675.90 | 2.1.5 |
| Subject 5 | Simulated DBS | 2 | 10 | 110 | PC | Qiagen | KHP | 308.38 | 2.1.5 |
| Subject 5 | Simulated DBS |  | 10 | 75 | PC | Qiagen | KHP | 300.00 | 2.1.5 |
| Subject 6 | Simulated DBS | 8 | 10 | 102 | FTA | Qiagen | KHP | 300.00 | 3.4.5 |
| Subject 6 | Simulated DBS | 10 | 10 | 75 | PC | Qiagen | KHP | 300.00 | 3.4.5 |
| Subject 6 | Simulated DBS |  | 10 | 89 | PC | Qiagen | KHP | 312.84 | 3.4.5 |
| Subject 7 | Simulated DBS | 2 | 10 | 60 | FTA | Qiagen | KHP | 565.20 | 2.1.5 |
| Subject 7 | Simulated DBS | 2 | 10 | 25 | PC | Qiagen | KHP | 300.00 | 2.1.5 |
| Subject 7 | Simulated DBS |  | 10 | 10 | PC | Qiagen | KHP | 300.00 | 2.1.5 |
| Subject 8 | Simulated DBS | 8 | 10 | 39 | FTA | Qiagen | KHP | 300.00 | 2.1.5 |
| Subject 8 | Simulated DBS | 10 | 10 | 18 | PC | Qiagen | KHP | 220.28 | 2.1.5 |
| Subject 9 | Simulated DBS | 8 | 10 | 36 | FTA | Qiagen | KHP | 519.00 | 2.1.5 |
| Subject 9 | Simulated DBS | 10 | 10 | 1 | PC | Qiagen | KHP | 300.00 | 2.1.6 |
| 1 | CDPH DBS | 1 | 6 | 167 | Whatman | Illumina | Illumina | 425.25 | 3.5.7 |
| 2 | CDPH DBS | 1 | 6 | 167 | Whatman | Illumina | Illumina | 500.00 | 3.5.7 |
| 3 | CDPH DBS | 1 | 6 | 180 | Whatman | Illumina | Illumina | 316.23 | 3.5.7 |
| 4 | CDPH DBS | 1 | 6 | 193 | Whatman | Illumina | Illumina | 485.00 | 3.5.7 |
| 5 | CDPH DBS | 1 | 6 | 524 | Whatman | Illumina | Illumina | 486.25 | 3.5.7 |
| 6 | CDPH DBS | 1 | 6 | 675 | Whatman | Illumina | Illumina | 498.65 | 3.5.7 |
| 7 | CDPH DBS | 1 | 6 | 2011 | Whatman | Illumina | Illumina | 258.00 | 3.5.7 |
| 8 | CDPH DBS | 1 | 6 | 2011 | Whatman | Illumina | Illumina | 267.75 | 3.5.7 |
| 9 | CDPH DBS | 1 | 6 | 2250 | Whatman | Illumina | Illumina | 358.80 | 3.5.7 |
| 10 | CDPH DBS | 1 | 6 | 2267 | Whatman | Illumina | Illumina | 460.85 | 3.5.7 |
| 11 | CDPH DBS | 1 | 6 | 2518 | PerkinElmer | Illumina | Illumina | 544.88 | 3.5.7 |
| 12 | CDPH DBS | 1 | 6 | 2536 | PerkinElmer | Illumina | Illumina | 500.00 | 3.5.7 |
| 13 | CDPH DBS | 1 | 6 | 2742 | PerkinElmer | Illumina | Illumina | 429.65 | 3.5.7 |
| 14 | CDPH DBS | 1 | 6 | 2742 | PerkinElmer | Illumina | Illumina | 500.00 | 3.5.7 |
| 15 | CDPH DBS | 1 | 6 | 3297 | Whatman | Illumina | Illumina | 473.78 | 3.5.7 |
| 16 | CDPH DBS | 1 | 6 | 3297 | Whatman | Illumina | Illumina | 454.75 | 3.5.7 |
| 17 | CDPH DBS | 1 | 6 | 4661 | Whatman | Illumina | Illumina | 361.38 | 3.5.7 |
| 18 | CDPH DBS | 1 | 6 | 4661 | Whatman | Illumina | Illumina | 500.00 | 3.5.7 |
| 19 | CDPH DBS | 1 | 6 | 4720 | Whatman | Illumina | Illumina | 433.73 | 3.5.7 |
| 20 | CDPH DBS | 1 | 6 | 4720 | Whatman | Illumina | Illumina | 377.70 | 3.5.7 |
| 21 | CDPH DBS | 1 | 6 | 5862 | Unknown | Illumina | Illumina | 268.00 | 3.5.7 |
| 22 | CDPH DBS | 1 | 6 | 5988 | Unknown | Illumina | Illumina | 475.20 | 3.5.7 |
| 23 | CDPH DBS | 1 | 6 | 5971 | Unknown | Illumina | Illumina | 346.60 | 3.5.7 |
| 24 | CDPH DBS | 1 | 6 | 6429 | Unknown | Illumina | Illumina | 500.00 | 3.5.7 |
| 25 | CDPH DBS | 1 | 6 | 6429 | Unknown | Illumina | Illumina | 500.00 | 3.5.7 |
| 26 | CDPH DBS | 1 | 6 | 6524 | Unknown | Illumina | Illumina | 500.00 | 3.5.7 |
| 27 | CDPH DBS | 1 | 6 | 6524 | Unknown | Illumina | Illumina | 500.00 | 3.5.7 |
| 28 | CDPH DBS | 1 | 6 | 7516 | Unknown | Illumina | Illumina | 500.00 | 3.5.7 |
| 29 | CDPH DBS | 1 | 6 | 7516 | Unknown | Illumina | Illumina | 500.00 | 3.5.7 |

**Supplementary Table 2: Quantity and quality of genomic DNA and sequencing library concentration of 63 manufactured DBS from 25 individuals, utilizing two types of filter papers, compared with matched blood samples.** NA: not applicable.

| **Subject** |  | **FTA Dried Blood Spot** | | | | **Protein Saver Dried Blood Spot** | | | | **350 µl Blood Sample** | | | |
| --- | --- | --- | --- | --- | --- | --- | --- | --- | --- | --- | --- | --- | --- |
|  | **Library Preparation Method** | **DNA yield (ng)** | **A260/ A280 ratio** | **Library input DNA (ng)** | **Library yield (nM)** | **DNA yield (ng)** | **A260/ A280 ratio** | **Library input DNA (ng)** | **Library yield (nM)** | **DNA yield (ng)*** | **A260/ A280 ratio** | **Library input DNA (ng)** | **Library yield (nM)** |
| 1 | Illumina | 285 | 1.59 | 268 | 7.7 | 263 | 1.54 | 247 | 8.8 | 6,670 | 1.85 | 1000 | 13.4 |
| 4 |  | 333 | 1.61 | 312 | 7.3 | 445 | 1.52 | 417 | 7.1 | 7,340 | 1.83 | 1000 | 14.4 |
| 10 |  | 180 | 1.48 | 169 | 5.9 | 225 | 1.44 | 211 | 6.9 | 5,332 | 1.78 | 1000 | 13.9 |
| 11_1 |  | 844 | 1.64 | 500 | 10.3 | 920 | 1.67 | 500 | 10.2 | 9,244 | 1.82 | 1000 | 19.2 |
| 13_1 |  | 761 | 1.77 | 500 | 8.4 | 674 | 1.82 | 500 | 8.1 | 13,130 | 1.84 | 1000 | 14.4 |
| 14 |  | 220 | 1.48 | 207 | 5.7 | 199 | 1.46 | 187 | 7.6 | 5,580 | 1.8 | 1000 | 27.3 |
| 18 |  | 262 | 1.42 | 246 | 5.9 | 322 | 1.46 | 302 | 7.1 | 5,096 | 1.8 | 1000 | 10.7 |
| 19 |  | 183 | 1.48 | 172 | 4.9 | 189 | 1.53 | 178 | 4.6 | 4,672 | 1.8 | 1000 | 11.8 |
| 20 |  | 190 | 1.6 | 179 | 6.6 | NA | NA | NA | NA | 7,200 | 1.71 | 1000 | 13.2 |
| 21 |  | 243 | 1.59 | 228 | 7.8 | NA | NA | NA | NA | 9,537 | 1.79 | 1000 | 20.3 |
| 22 |  | 229 | 1.7 | 215 | 6.6 | NA | NA | NA | NA | 6,046 | 1.65 | 1000 | 13.4 |
| 23 |  | 165 | 1.54 | 155 | 7 | NA | NA | NA | NA | 6,831 | 1.81 | 1000 | 15.3 |
| 24 |  | 204 | 1.53 | 191 | 6.6 | NA | NA | NA | NA | 9,521 | 1.82 | 1000 | 11.3 |
| 25 |  | 212 | 1.58 | 199 | 6.8 | NA | NA | NA | NA | 7,551 | 1.78 | 1000 | 15 |
| 11_2 |  | 302 | 1.59 | 283 | 5.9 | NA | NA | NA | NA | 9,244 | 1.82 | 1000 | 19.2 |
| 13_2 |  | 535 | 1.73 | 501 | 6.7 | NA | NA | NA | NA | 13,130 | 1.84 | 1000 | 14.4 |
| **Average** |  | **322** | **1.58** | **270** | **6.9** | **405** | **1.56** | **318** | **7.6** | **7,411** | **1.79** | **1000** | **15.3** |
| 1 | KAPA | 561 | 1.6 | 421 | 7.8 | 609 | 1.68 | 300 | 3 | 6,670 | 1.85 | 1000 | 13.4 |
| 2 |  | 1,338 | 1.75 | 1004 | 13.6 | 948 | 1.76 | 300 | 4.4 | 5,900 | 1.84 | 1000 | 12 |
| 3 |  | 984 | 1.76 | 738 | 9.2 | 1,728 | 1.72 | 300 | 4.8 | 10,240 | 1.84 | 1000 | 17.6 |
| 4 |  | 783 | 1.77 | 587 | 8.5 | 1,120 | 1.72 | 300 | 13.8 | 7,340 | 1.83 | 1000 | 14.4 |
| 5 |  | 901 | 1.77 | 676 | 5.6 | 987 | 1.72 | 308 | 3.7 | 5,190 | 1.79 | 1000 | 15.9 |
| 6 |  | 851 | 1.78 | 300 | 5.4 | 832 | 1.78 | 300 | 4.9 | 5,090 | 1.84 | 1000 | 15 |
| 7 |  | 754 | 1.75 | 565 | 6.1 | 694 | 1.76 | 300 | 6.4 | 5,260 | 1.85 | 1000 | 13.2 |
| 8 |  | 400 | 1.75 | 300 | 5.8 | 441 | 1.67 | 220 | 7.5 | 4,767 | 1.73 | 1000 | 13.8 |
| 9 |  | 692 | 1.63 | 519 | 7.3 | 792 | 1.53 | 300 | 6.6 | 6,115 | 1.78 | 1000 | 11.3 |
| 10 |  | 642 | 1.61 | 482 | 11.8 | 430 | 1.6 | 300 | 3.1 | 5,332 | 1.78 | 1000 | 13.9 |
| 11 |  | 1,394 | 1.72 | 523 | 18.8 | 1,638 | 1.76 | 300 | 7.1 | 9,244 | 1.82 | 1000 | 19.2 |
| 12 |  | 762 | 1.71 | 571 | 11.6 | 756 | 1.76 | 300 | 6.1 | 7,851 | 1.99 | 1000 | 23.9 |
| 13 |  | NA | NA | NA | NA | 1,312 | 1.82 | 300 | 15.9 | 13,130 | 1.84 | 1000 | 14.4 |
| 14 |  | NA | NA | NA | NA | 417 | 1.61 | 300 | 7.1 | 5,580 | 1.8 | 1000 | 27.3 |
| 15 |  | 409 | 1.66 | 300 | 7.2 | 624 | 1.66 | 300 | 7.1 | 5,356 | 1.83 | 1000 | 13.3 |
| 16 |  | NA | NA | NA | NA | 369 | 1.6 | 277 | 7.3 | 6,200 | 1.81 | 1000 | 11.8 |
| 17 |  | NA | NA | NA | NA | 373 | 1.77 | 280 | 12.2 | 6,370 | 1.8 | 1000 | 12.8 |
| **Average** |  | **806** | **1.71** | **537** | **9.1** | **828** | **1.7** | **293** | **7.1** | **6,583** | **1.83** | **1000** | **15.3** |
| 1 | KAPA |  |  |  |  | 702 | 1.83 | 527 | 7.4 | 6,670 | 1.85 | 1000 | 13.4 |
| 2 |  |  |  |  |  | 1,137 | 1.88 | 853 | 6.8 | 5,900 | 1.84 | 1000 | 12 |
| 3 |  |  |  |  |  | 1,971 | 1.8 | 370 | 5.1 | 10,240 | 1.84 | 1000 | 17.6 |
| 5 |  |  |  |  |  | 912 | 1.73 | 300 | 10.5 | 5,190 | 1.79 | 1000 | 15.9 |
| 6 |  |  |  |  |  | 1,251 | 1.79 | 313 | 6.2 | 5,090 | 1.84 | 1000 | 15 |
| 7 |  |  |  |  |  | 651 | 1.8 | 300 | 7.1 | 5,260 | 1.85 | 1000 | 13.2 |
| 10 |  |  |  |  |  | 452 | 1.65 | 339 | 13.2 | 5,332 | 1.78 | 1000 | 13.9 |
| 11 |  |  |  |  |  | 1,524 | 1.78 | 381 | 4.9 | 9,244 | 1.82 | 1000 | 19.2 |
| 12 |  |  |  |  |  | 715 | 1.72 | 357 | 4.5 | 7,851 | 1.99 | 1000 | 23.9 |
| **Average** |  |  |  |  |  | **1,035** | **1.78** | **416** | **7.3** | **6,753** | **1.84** | **1000** | **16** |

**Supplementary Table 3:** **Quantity and quality of WGS derived from manufactured DBS and matched blood samples with two different library preparation methods (Illumina and KAPA) and three different sequencing flow cells.** Abbreviations: Q: quality score.

|  | **Library Preparation Method** | **Flowcell type** | **Number of flowcells used** | **Number of Samples for which WGS performed** | **Read Length (nt)** | **Total yield per flowcell (Gb)** | **Error rate (%)** | **% of Clusters passing filters** | **% of called bases with >Q30** | **% with Correct Index** |
| --- | --- | --- | --- | --- | --- | --- | --- | --- | --- | --- |
| **DBS research WGS** | **KAPA** | S1 | 15 | 36 | 2x101 | 415 | 0.2 | 75.3 | 93.7 | 94.0 |
|  |  | S2 | 10 | 49 | 2x101 | 947 | 0.2 | 76.8 | 92.9 | 93.8 |
|  | **Illumina** | S4 | 1 | 24 | 2x101 | 3270 | 0.2 | 67.2 | 89.5 | 86.0 |
| **Clinical grade WGS (blood samples)** | **KAPA** | S1 | 8 | 20 | 2x101 | 444 | 0.2 | 80.8 | 94.1 | 95.3 |
|  |  | S2 | 5 | 28 | 2x101 | 977 | 0.2 | 78.7 | 92.9 | 95.1 |
|  | **Illumina** | S4 | 2 | 44 | 2x151 | 3340 | 0.3 | 69.2 | 87.8 | 91.1 |

**Supplementary Table 4: Quality metrics of aligned WGS reads from Illumina and KAPA libraries prepared from manufactured DBS.** Abbreviations: DBS: dried blood spot; CD: coding domain; OMIM: Mendelian inheritance in Man; MT: mitochondrial; SNV: single nucleotide variant; indel: insertion-deletion oligonucleotide variant; CNV: copy number variant.

| **Library Prep** | **Sample ID** | **Raw Yield (Gb)** | **% Reads Align-ed** | **% Duplicate Reads** | **Mean Insert Size (nt)** | **Average Genome Coverage** | **% MIM genes with >10X coverage of 100% of CD** | **Coding Domain Variants** | **SNVs** | **Indels** | **CNVs Overlapping MIM gene coding domains** | **Mitoch-ondrial genome coverage** | **Trans-ition/ Trans-version ratio** | **C>T+ A>G/ T>C+ G>A SNP variant ratio** | **Pro-port-ion-ate GC bias** | **Total Length of Mapp-able Genome (Gb)** |
| --- | --- | --- | --- | --- | --- | --- | --- | --- | --- | --- | --- | --- | --- | --- | --- | --- |
| Illumina | 1 | 167 | 97.7% | 9.4% | 466 | 45.2 | 98.2% | 24,499 | 3,976,208 | 940,505 | 3 | 3,419 | 2.03 | 1.04 | -0.09 | ND |
|  | 4 | 151 | 97.8% | 9.0% | 456 | 41.2 | 96.9% | 24,141 | 3,918,968 | 928,905 | 2 | 4,509 | 2.03 | 1.05 | -0.04 | ND |
|  | 10 | 137 | 97.9% | 15.6% | 422 | 36 | 93.9% | 24,989 | 3,826,902 | 951,538 | 18 | 1,714 | 2.03 | 1.05 | -0.07 | ND |
|  | 11_1 | 118 | 97.7% | 15.1% | 472 | 31.2 | 93.2% | 26,115 | 3,970,638 | 979,030 | 13 | 3,167 | 2.04 | 1.05 | -0.07 | 2.67 |
|  | 13_1 | 120 | 97.8% | 8.4% | 429 | 33.1 | 96.9% | 29,540 | 4,768,773 | 1,094,754 | 11 | 2,451 | 2.04 | 1.05 | -0.02 | ND |
|  | 14 | 145 | 97.8% | 15.6% | 412 | 38.1 | 94.1% | 24,942 | 3,838,925 | 955,966 | 12 | 2,194 | 2.03 | 1.05 | -0.02 | ND |
|  | 18 | 128 | 97.7% | 13.0% | 428 | 34.8 | 96.1% | 25,446 | 3,895,926 | 970,452 | 10 | 2,544 | 2.03 | 1.05 | -0.23 | ND |
|  | 19 | 125 | 97.7% | 13.4% | 441 | 33.8 | 92.8% | 25,342 | 3,835,352 | 954,747 | 14 | 2,166 | 2.03 | 1.05 | -0.14 | ND |
|  | 20 | 155 | 97.8% | 13.7% | 356 | 41.6 | 95.4% | 25,117 | 3,831,970 | 957,613 | 11 | 2,928 | 2.03 | 1.04 | 0.00 | ND |
|  | 21 | 159 | 98.1% | 13.2% | 368 | 43.3 | 97.3% | 24,961 | 3,844,408 | 961,100 | 10 | 8,310 | 2.03 | 1.04 | 0.01 | 2.67 |
|  | 22 | 149 | 98.0% | 13.5% | 378 | 40.3 | 94.8% | 24,823 | 3,803,526 | 948,193 | 16 | 3,778 | 2.03 | 1.04 | -0.16 | ND |
|  | 23 | 140 | 98.1% | 9.1% | 367 | 39.7 | 93.6% | 25,286 | 3,913,982 | 975,878 | 20 | 3,870 | 2.03 | 1.05 | 0.13 | ND |
|  | 24 | 135 | 97.9% | 14.5% | 386 | 36 | 92.3% | 25,249 | 3,861,366 | 959,910 | 7 | 3,059 | 2.03 | 1.05 | 0.10 | ND |
|  | 25 | 153 | 98.0% | 13.4% | 376 | 41.4 | 96.0% | 25,258 | 3,828,696 | 958,608 | 10 | 6,523 | 2.03 | 1.05 | 0.09 | ND |
|  | 11_2 | 146 | 97.9% | 12.8% | 409 | 39.7 | 95.7% | 25,991 | 3,968,906 | 986,333 | 14 | 4,811 | 2.03 | 1.04 | -0.31 | 2.67 |
|  | 13_2 | 140 | 97.9% | 8.6% | 400 | 40 | 96.0% | 25,074 | 3,831,223 | 957,676 | 15 | 2,527 | 2.03 | 1.05 | -0.05 | ND |
|  | 1 | 151 | 97.7% | 8.5% | 482 | 41.5 | 98.3% | 24,533 | 3,974,243 | 937,622 | 4 | 2,675 | 2.03 | 1.04 | -0.09 | ND |
|  | 4 | 124 | 97.2% | 8.7% | 465 | 33.6 | 94.9% | 24,156 | 3,908,774 | 914,642 | 2 | 2,675 | 2.03 | 1.04 | -0.18 | ND |
|  | 10 | 131 | 98.0% | 15.3% | 437 | 34.6 | 92.5% | 25,004 | 3,828,786 | 950,945 | 45 | 777 | 2.03 | 1.04 | -0.13 | ND |
|  | 11_1 | 144 | 97.8% | 13.6% | 442 | 38.7 | 95.6% | 26,013 | 3,971,201 | 986,218 | 12 | 4,139 | 2.03 | 1.04 | -0.07 | ND |
|  | 13_1 | 124 | 97.8% | 15.7% | 444 | 32.6 | 96.3% | 30,435 | 4,670,651 | 1,135,905 | 16 | 3,666 | 2.03 | 1.05 | 0.08 | 2.67 |
|  | 14 | 127 | 97.9% | 14.7% | 435 | 34 | 93.0% | 25,023 | 3,841,870 | 953,906 | 10 | 4,171 | 2.03 | 1.05 | 0.10 | 2.67 |
|  | 18 | 117 | 97.7% | 13.0% | 444 | 31.7 | 91.9% | 25,417 | 3,897,828 | 968,605 | 11 | 4,014 | 2.03 | 1.05 | 0.01 | 2.67 |
|  | 19 | 151 | 97.6% | 14.9% | 442 | 40 | 95.2% | 25,252 | 3,831,281 | 955,401 | 10 | 2,657 | 2.03 | 1.05 | 0.06 | 2.67 |
| Avg. |  | 139 | 97.8% | 12.6% | 423 | 37.6 | 95.0% | 25,525 | 3,951,683 | 970,186 | 12 | 3,448 | 2.03 | 1.05 | -0.04 | 2.67 |
| SD |  | 14 | 0.2% | 2.6% | 36.1 | 4.0 | 1.9% | 1,467 | 243,712 | 48,051 | 8.42 | 1,561 | 0.00 | 0.00 | 0.11 | 0.00 |
| Median |  | 140 | 97.8% | 13.4% | 432 | 38.4 | 95.3% | 25,183 | 3,878,646 | 957,645 | 11 | 3,113 | 2.03 | 1.05 | -0.05 | 2.67 |
| KAPA | 1 | 150 | 98.5% | 10.2% | 355 | 42.2 | 90.8% | 24,624 | 3,796,486 | 924,123 | 3399 | 7,161 | 2.04 | **1.04** | 0.78 | 2.67 |
|  | 2 | 134 | 98.4% | 10.5% | 322 | 37.7 | 96.9% | 30,061 | 4,629,653 | 1,109,978 | 19 | 3,053 | 2.04 | **1.05** | 0.14 | 2.67 |
|  | 3 | 153 | 98.1% | 11.6% | 166 | 42.3 | 92.0% | 24,760 | 3,809,095 | 925,033 | 7 | 45,617 | 2.01 | 1.05 | 0.05 | 2.67 |
|  | 4 | 141 | 98.7% | 11.0% | 338 | 37.7 | 87.1% | 23,460 | 3,877,648 | 907,633 | 802 | 3,414 | 2.04 | 1.05 | -0.14 | 2.67 |
|  | 5 | 159 | 98.2% | 9.7% | 340 | 44.9 | 94.9% | 25,642 | 3,876,005 | 952,271 | 9 | 6,585 | 2.02 | 1.05 | -0.16 | 2.67 |
|  | 6 | 138 | 98.3% | 10.0% | 308 | 38.8 | 96.9% | 24,775 | 3,823,390 | 933,632 | 19 | 3,686 | 2.04 | 1.05 | -0.02 | 2.66 |
|  | 7 | 139 | 98.2% | 10.7% | 324 | 38.7 | 94.5% | 24,950 | 3,793,367 | 929,337 | 57 | 4,355 | 2.04 | 1.04 | 0.15 | 2.67 |
|  | 8 | 126 | 98.4% | 10.9% | 312 | 35.3 | 95.9% | 25,077 | 3,833,349 | 937,397 | 5 | 4,252 | 2.03 | 1.05 | 0.08 | 2.66 |
|  | 9 | 222 | 98.3% | 10.7% | 327 | 59.1 | 97.5% | 24,674 | 3,910,898 | 922,371 | 11 | 8,494 | 2.04 | 1.05 | -0.09 | 2.66 |
|  | 10 | 123 | 98.2% | 9.3% | 329 | 34.9 | 93.5% | 24,920 | 3,788,664 | 927,058 | 19 | 4,514 | 2.01 | 1.05 | -0.06 | 2.67 |
|  | 11 | 159 | 98.4% | 11.3% | 374 | 44.1 | 95.5% | 28,137 | 4,313,297 | 1,041,608 | 324 | 8,045 | 2.02 | 1.05 | -0.19 | 2.67 |
|  | 12 | 126 | 98.3% | 10.1% | 338 | 35.4 | 93.5% | 24,826 | 3,799,191 | 930,170 | 95 | 3,837 | 2.04 | 1.05 | 0.17 | 2.67 |
|  | 13 | 168 | 98.7% | 13.4% | 477 | 45.9 | 96.5% | 25,306 | 3,858,340 | 1,000,164 | 122 | 2,809 | 1.98 | 1.04 | 0.13 | 2.67 |
|  | 14 | 128 | 98.3% | 10.7% | 277 | 33.7 | 90.8% | 23,914 | 3,875,802 | 889,363 | 24 | 5,517 | 2.01 | 1.05 | 0.03 | 2.67 |
|  | 15 | 191 | 98.0% | 12.6% | 360 | 49.4 | 96.6% | 24,262 | 3,908,065 | 910,914 | 8 | 7,107 | 2.03 | 1.05 | -0.06 | 2.67 |
|  | 16 | 124 | 98.2% | 10.7% | 267 | 34.7 | 92.8% | 30,024 | 4,620,648 | 1,106,103 | 24 | 4,692 | 2.04 | 1.05 | 0.04 | 2.66 |
|  | 17 | 151 | 98.2% | 11.1% | 176 | 42.1 | 95.9% | 29,805 | 4,597,642 | 1,093,977 | 15 | 5,208 | 2.04 | 1.05 | 0.08 | 2.66 |
|  | 1 | 186 | 98.6% | 11.9% | 392 | 49.1 | 93.2% | 24,192 | 3,890,452 | 918,085 | 455 | 4,377 | 2.02 | 1.05 | -0.07 | 2.67 |
|  | 2 | 152 | 98.6% | 10.8% | 291 | 40.3 | 94.4% | 24,278 | 3,885,525 | 899,205 | 11 | 2,763 | 2.02 | 1.05 | -0.16 | 2.67 |
|  | 3 | 164 | 98.3% | 10.0% | 366 | 44 | 80.3% | 24,334 | 3,925,503 | 920,647 | 1036 | 41,267 | 2.02 | 1.05 | 0.02 | 2.67 |
|  | 4 | 166 | 98.4% | 10.7% | 282 | 44 | 95.6% | 24,676 | 3,930,421 | 914,413 | 6 | 30,749 | 2.04 | 1.04 | -0.04 | 2.67 |
|  | 5 | 151 | 98.2% | 9.7% | 258 | 40.5 | 94.9% | 25,224 | 4,028,693 | 935,185 | 527 | 6,741 | 2.03 | 1.05 | 0.56 | 2.67 |
|  | 6 | 175 | 98.6% | 11.4% | 379 | 46.6 | 85.4% | 24,393 | 3,962,043 | 931,888 | 868 | 11,552 | 2.03 | 1.05 | -0.12 | 2.67 |
|  | 7 | 133 | 98.4% | 10.5% | 301 | 35.4 | 97.1% | 24,324 | 3,885,962 | 904,657 | 2 | 3,610 | 2.03 | 1.05 | -0.25 | 2.67 |
|  | 8 | 246 | 98.2% | 12.5% | 287 | 63.7 | 97.8% | 23,857 | 3,905,492 | 921,577 | 12 | 6,436 | 2.03 | 1.05 | 0.11 | 2.67 |
|  | 9 | 251 | 97.7% | 11.8% | 179 | 64.8 | 97.2% | 24,737 | 3,913,059 | 916,348 | 2 | 10,632 | 2.03 | 1.04 | 0.25 | 2.66 |
|  | 10 | 190 | 98.2% | 11.9% | 314 | 49.6 | 94.8% | 25,057 | 3,967,099 | 933,370 | 66 | 6,353 | 2.02 | 1.04 | -0.09 | 2.67 |
|  | 11 | 161 | 97.8% | 12.3% | 285 | 44 | 94.4% | 25,709 | 3,866,656 | 944,885 | 4 | 5,388 | 2.04 | 1.05 | 0.35 | 2.67 |
|  | 12 | 183 | 98.5% | 11.7% | 300 | 48 | 97.4% | 24,313 | 3,922,361 | 919,166 | 32 | 4,345 | 2.03 | 1.05 | 0.15 | 2.67 |
|  | 13 | 142 | 98.8% | 11.0% | 302 | 37.9 | 97.0% | 24,376 | 3,920,145 | 903,814 | 7 | 3,126 | 2.04 | 1.04 | -0.04 | 2.67 |
|  | 14 | 143 | 98.2% | 9.3% | 207 | 38.3 | 94.2% | 24,416 | 3,877,255 | 899,806 | 351 | 4,925 | 2.03 | 1.05 | 0.32 | 2.66 |
|  | 15 | 150 | 98.3% | 11.1% | 278 | 39.4 | 96.9% | 24,344 | 3,912,332 | 910,758 | 61 | 4,403 | 2.03 | 1.05 | 0.06 | 2.67 |
|  | 16 | 177 | 98.0% | 10.5% | 228 | 46.7 | 97.2% | 24,498 | 3,917,575 | 906,083 | 3 | 5,104 | 2.03 | 1.05 | 0.03 | 2.67 |
|  | 17 | 127 | 98.1% | 10.6% | 321 | 33.6 | 81.2% | 24,233 | 3,873,067 | 888,270 | 159 | 3,444 | 2.02 | 1.05 | -0.03 | 2.67 |
|  | 1 | 160 | 97.7% | 10.3% | 247 | 42 | 95.1% | 24,564 | 3,891,727 | 901,304 | 5 | 4,724 | 2.03 | 1.05 | -0.19 | 2.67 |
|  | 2 | 158 | 98.6% | 11.5% | 376 | 41.9 | 94.2% | 24,228 | 3,891,372 | 912,330 | 145 | 5,812 | 2.02 | 1.05 | 0.08 | 2.67 |
|  | 3 | 159 | 98.4% | 10.6% | 384 | 44.6 | 90.5% | 24,832 | 3,779,735 | 925,516 | 271 | 5,524 | 2.04 | 1.05 | 0.23 | 2.67 |
|  | 5 | 220 | 98.2% | 12.2% | 196 | 57.2 | 97.3% | 27,760 | 4,426,478 | 1,027,196 | 8 | 9,986 | 2.03 | 1.05 | 0.00 | 2.67 |
|  | 6 | 135 | 98.3% | 11.0% | 373 | 35.9 | 81.3% | 24,060 | 3,885,219 | 909,090 | 249 | 4,920 | 2.02 | 1.05 | 0.02 | 2.67 |
| Avg. |  | 160 | 98.3% | 11.0% | 306 | 43.2 | 93.6% | 25,170 | 3,963,326 | 940,634 | 237 | 8,065 | 2.03 | 1.05 | 0.05 | 2.67 |
| SD |  | 32 | 0.3% | 0.9% | 66.3 | 7.7 | 4.6% | 1,654 | 226,097 | 57,237 | 580 | 9,504 | 0.01 | 0.00 | 0.20 | 0.00 |
| Median |  | 153 | 98.3% | 10.8% | 312 | 42.1 | 94.9% | 24,674 | 3,891,372 | 922,371 | 24 | 5,104 | 2.03 | 1.05 | 0.03 | 2.67 |
| KAPA | 1 | 200.3 | 98.7% | 19.20% | 409.0 | 48.5 | 96.84% | 24,346 | 3,916,830 | 916,832 | 3 | nd | 2.03 | 1.047 | -0.07 | 2.67 |
|  | 2 | 163.0 | 98.4% | 14.80% | 394.8 | 41.4 | 95.97% | 25,187 | 3,975,940 | 923,041 | 4 | nd | 2.03 | 1.048 | 0.04 | 2.67 |
|  | 3 | 136.5 | 98.8% | 9.60% | 408.8 | 37.1 | 97.69% | 24,352 | 3,933,824 | 896,828 | 14 | nd | 2.02 | 1.048 | 0.12 | 2.67 |
|  | 4 | 161.0 | 98.6% | 11.00% | 381.3 | 43.0 | 96.92% | 24,659 | 3,908,034 | 910,782 | 31 | nd | 2.03 | 1.047 | 0.18 | 2.67 |
|  | 5 | 189.7 | 98.8% | 10.50% | 382.3 | 51.2 | 98.36% | 24,718 | 3,946,106 | 929,291 | 16 | nd | 2.02 | 1.047 | 0.17 | 2.67 |
|  | 6 | 142.1 | 98.5% | 8.60% | 415.6 | 38.8 | 96.38% | 24,658 | 3,903,231 | 901,676 | 9 | nd | 2.03 | 1.047 | 0.15 | 2.67 |
|  | 7 | 190.5 | 98.7% | 12.70% | 400.1 | 49.9 | 97.83% | 24,669 | 3,914,092 | 917,267 | 95 | nd | 2.02 | 1.047 | 0.24 | 2.67 |
|  | 8 | 170.7 | 98.7% | 10.60% | 395.6 | 45.8 | 97.20% | 27,828 | 4,449,910 | 1,029,642 | 10 | nd | 2.03 | 1.053 | 0.12 | 2.67 |
|  | 9 | 163.6 | 98.6% | 10.70% | 386.4 | 43.8 | 97.05% | 24,434 | 3,909,694 | 921,106 | 19 | nd | 2.02 | 1.042 | 0.18 | 2.67 |
|  | 10 | 173.0 | 98.7% | 9.50% | 415.1 | 47.1 | 98.14% | 29,575 | 4,746,463 | 1,081,171 | 13 | nd | 2.03 | 1.055 | 0.08 | 2.67 |
|  | 11 | 179.8 | 98.7% | 13.60% | 415.8 | 46.7 | 97.78% | 24,705 | 3,934,453 | 916,316 | 4 | nd | 2.01 | 1.047 | 0.07 | 2.67 |
|  | 12 | 174.6 | 98.7% | 12.30% | 299.5 | 45.9 | 97.15% | 24,823 | 3,941,611 | 920,065 | 10 | nd | 2.02 | 1.048 | 0.08 | 2.67 |
|  | 13 | 194.3 | 98.5% | 14.60% | 391.2 | 49.5 | 97.66% | 25,483 | 4,056,403 | 947,695 | 17 | nd | 2.03 | 1.048 | 0.11 | 2.67 |
|  | 14 | 240.5 | 98.8% | 10.40% | 308.0 | 64.9 | 98.31% | 24,711 | 3,980,954 | 940,746 | 7 | nd | 2.03 | 1.046 | -0.02 | 2.67 |
|  | 15 | 178.5 | 98.6% | 9.80% | 454.6 | 48.3 | 97.92% | 24,581 | 3,912,119 | 903,558 | 3 | nd | 2.02 | 1.046 | 0.02 | 2.67 |
|  | 16 | 186.7 | 98.5% | 9.90% | 468.8 | 50.4 | 97.94% | 24,030 | 3,911,614 | 901,162 | 12 | nd | 2.02 | 1.046 | 0.04 | 2.67 |
|  | 17 | 200.9 | 98.7% | 13.30% | 452.4 | 52.3 | 97.92% | 25,009 | 3,948,859 | 926,984 | 7 | nd | 2.02 | 1.050 | 0.14 | 2.67 |
|  | 18 | 150.2 | 98.7% | 9.00% | 430.7 | 41.1 | 97.96% | 24,274 | 3,934,929 | 903,469 | 2 | nd | 2.03 | 1.046 | 0.00 | 2.67 |
|  | 19 | 148.5 | 98.4% | 9.20% | 418.9 | 40.3 | 95.91% | 24,024 | 3,874,838 | 888,758 | 7 | nd | 2.03 | 1.047 | 0.07 | 2.67 |
|  | 20 | 178.8 | 98.2% | 11.80% | 432.0 | 46.9 | 97.21% | 24,427 | 3,906,648 | 902,079 | 8 | nd | 2.03 | 1.046 | -0.09 | 2.67 |
|  | 21 | 143.6 | 98.6% | 11.20% | 443.1 | 38.2 | 97.67% | 24,259 | 3,907,928 | 893,522 | 4 | nd | 2.03 | 1.046 | 0.05 | 2.67 |
|  | 22 | 205.0 | 98.5% | 8.40% | 458.7 | 56.2 | 98.25% | 24,247 | 3,883,571 | 906,849 | 9 | nd | 2.02 | 1.047 | -0.01 | 2.67 |
|  | 23 | 154.5 | 98.5% | 7.10% | 412.4 | 43.0 | 97.79% | 24,300 | 3,978,708 | 913,144 | 17 | nd | 2.03 | 1.046 | 0.02 | 2.67 |
|  | 24 | 133.9 | 98.5% | 8.70% | 435.8 | 36.7 | 94.87% | 24,339 | 3,924,305 | 890,949 | 26 | nd | 2.03 | 1.049 | 0.00 | 2.67 |
|  | 25 | 170.7 | 98.5% | 8.70% | 386.1 | 46.6 | 97.75% | 24,501 | 3,908,433 | 906,768 | 5 | nd | 2.03 | 1.049 | 0.00 | 2.67 |
| Avg. |  | 173.2 | 98.6% | 11.01% | 407.9 | 46.1 | 97.38% | 24,886 | 3,984,380 | 923,588 | 14 | nd | 2.03 | 1.047 | 0.07 | 2.67 |
| Median |  | 173.0 | 98.6% | 10.50% | 412.4 | 46.6 | 97.69% | 24,581 | 3,924,305 | 913,144 | 9 | nd | 2.03 | 1.047 | 0.07 | 2.67 |

**Supplementary Table 5: Supplementary Table 5: Quality control metrics for secondary analysis of WGS from DBS and blood.** Abbreviations: DBS: dried blood spot; CD: coding domain; MIM: Mendelian inheritance in Man; SNV: single nucleotide variant; indel: insertion-deletion oligonucleotide variant; CNV: copy number variant; Avg.: Average; Med.: Median; Cov.: Coverage.

| **Method** | **QC metrics** | **Raw Yield (Gb)** | **Reads Map-ped** | **Dupli- cate Reads** | **Align-ed Yield (Gb)** | **Mean insert size (nt)** | **Avg. Cov.** | **Avg. Cov. of MIM genes** | **% MIM genes with 100% CD Cov. >10X** | **Total coding variants** | **Total SNVs** | **Total Indels** | **CNVs Overlap-ping MIM gene CDs** | **Mito-chond-rial Genome Cov.** | **Sample size** |
| --- | --- | --- | --- | --- | --- | --- | --- | --- | --- | --- | --- | --- | --- | --- | --- |
| DBS Illumina | Avg. | 139.0 | 97.8% | 12.6% | 136.0 | 423 | 37.6 | 36.0 | 95.03% | 25,525 | 3,951,683 | 970186 | 12 | 3448 | 24 |
|  | Med. | 140.1 | 97.8% | 13.4% | 137.3 | 432 | 38.4 | 35.3 | 95.31% | 25,183 | 3,878,646 | 957645 | 11 | 3113 | 24 |
| DBS KAPA | Avg. | 160.5 | 98.3% | 11.0% | 157.7 | 306 | 43.2 | 42.6 | 93.56% | 25,170 | 3,963,326 | 940634 | 237 | 8197 | 39 |
|  | Med. | 153.1 | 98.3% | 10.8% | 150.2 | 312 | 42.1 | 41.5 | 94.87% | 24,674 | 3,891,372 | 922371 | 24 | 5156 | 39 |
| Blood Illumina | Avg. | 147.9 | 99.0% | 11.8% | 145.8 | 422 | 41.0 | 41.1 | 95.39% | 25,255 | 3,862,520 | 948,453 | 23 | 10598 | 24 |
|  | Med. | 142.7 | 99.1% | 11.9% | 141.3 | 424 | 40.2 | 40.9 | 95.62% | 25,054 | 3,834,070 | 942,348 | 11 | 10562 | 24 |
| Blood KAPA | Avg. | 160.9 | 98.6% | 10.8% | 158.6 | 383 | 43.0 | 45.0 | 96.63% | 25,078 | 3,996,783 | 931,744 | 22 | 10820 | 48 |
|  | Med. | 157.4 | 98.6% | 10.8% | 154.1 | 387 | 41.8 | 42.4 | 97.39% | 24,800 | 3,935,857 | 920,021 | 8 | 5152 | 48 |

**Supplementary Table 6: Quality metrics of aligned WGS reads from Illumina libraries prepared from California State Biobank DBS.** Abbreviations: DBS: dried blood spot; OMIM: Mendelian inheritance in Man; Nk: not known.

| **ID** | **Year Collected** | **DBS Paper Type** | **# of Punches avail-able** | **DNA yield (ng)** | **A260/ A280 ratio** | **Library input DNA (ng)** | **Library yield (nM)** | **Raw WGS Yield (GB)** | **% Reads Map-ped** | **% Dupli-cate Reads** | **Mean Insert Size (bp)** | **Avg. Genome Cover-age** | **Avg. Cover-age of MIM genes** | **% MIM genes with >10X cover-age of 100% coding domain** | **Prop-ortion-ate GC bias** |
| --- | --- | --- | --- | --- | --- | --- | --- | --- | --- | --- | --- | --- | --- | --- | --- |
| 1 | 2020 | Whatman | 10 | 544 | 1.77 | 425 | 8.0 | 155 | 99.1% | 12.9% | 433 | 42.8 | 40.3 | 97.2% | 0.01 |
| 2 | 2020 | Whatman | 10 | 689 | 1.81 | 500 | 7.8 | 123 | 99.1% | 11.4% | 437 | 34.3 | 32.4 | 95.0% | -0.03 |
| 3 | 2020 | Whatman | 10 | 405 | 1.6 | 316 | 10.3 | 108 | 99.3% | 8.3% | 280 | 31.4 | 30.6 | 92.4% | -0.06 |
| 4 | 2020 | Whatman | 12 | 621 | 1.67 | 485 | 10.0 | 130 | 99.3% | 8.4% | 443 | 37.6 | 37.7 | 97.6% | 0.10 |
| 5 | 2019 | Whatman | 8 | 622 | 1.64 | 486 | 7.0 | 158 | 99.3% | 8.9% | 443 | 45.6 | 44.9 | 97.0% | 0.10 |
| 6 | 2018 | Whatman | 12 | 638 | 1.73 | 499 | 13.5 | 114 | 99.4% | 8.7% | 406 | 33 | 32.4 | 93.6% | 0.09 |
| 7 | 2015 | Whatman | 12 | 331 | 1.83 | 258 | 13.1 | 97 | 99.4% | 8.4% | 249 | 28 | 29.5 | 90.8% | 0.07 |
| 8 | 2015 | Whatman | 8 | 343 | 1.7 | 268 | 6.8 | 149 | 99.3% | 8.4% | 337 | 43.3 | 42.3 | 97.6% | -0.16 |
| 9 | 2014 | Whatman | 12 | 459 | 1.7 | 359 | 10.2 | 142 | 99.3% | 7.8% | 436 | 41.5 | 41.2 | 96.0% | 0.03 |
| 10 | 2014 | Whatman | 12 | 590 | 1.87 | 461 | 10.7 | 101 | 99.4% | 9.3% | 430 | 29 | 26 | 75.1% | -0.21 |
| 11 | 2013 | PerkinElmer | 12 | 697 | 1.71 | 545 | 11.2 | 195 | 99.3% | 8.4% | 453 | 56.6 | 56.7 | 98.0% | 0.13 |
| 12 | 2013 | PerkinElmer | 12 | 934 | 1.73 | 500 | 12.2 | 158 | 99.0% | 13.8% | 433 | 42.9 | 39.6 | 95.2% | 0.03 |
| 13 | 2013 | PerkinElmer | 10 | 550 | 1.82 | 430 | 8.0 | 97 | 99.4% | 9.4% | 427 | 27.8 | 25 | 71.4% | -0.19 |
| 14 | 2013 | PerkinElmer | 10 | 769 | 2.11 | 500 | 6.3 | 133 | 99.3% | 7.7% | 462 | 38.7 | 37.9 | 95.5% | 0.02 |
| 15 | 2011 | Whatman | 10 | 606 | 1.66 | 474 | 7.0 | 115 | 99.3% | 8.0% | 427 | 33.6 | 29.3 | 78.5% | -0.37 |
| 16 | 2011 | Whatman | 10 | 582 | 1.72 | 455 | 7.0 | 114 | 99.3% | 8.4% | 420 | 33 | 28.6 | 76.7% | -0.38 |
| 17 | 2007 | Whatman | 6 | 925 | 1.78 | 361 | 7.0 | 143 | 99.1% | 13.5% | 430 | 39 | 36.2 | 94.1% | 0.00 |
| 18 | 2007 | Whatman | 7 | 787 | 1.8 | 500 | 6.6 | 148 | 99.1% | 13.2% | 443 | 40.7 | 38.2 | 94.6% | 0.03 |
| 19 | 2007 | Whatman | 8 | 555 | 1.57 | 434 | 8.2 | 101 | 99.3% | 7.6% | 435 | 29.5 | 25.8 | 69.9% | -0.34 |
| 20 | 2007 | Whatman | 10 | 483 | 1.52 | 378 | 7.9 | 146 | 99.2% | 10.1% | 427 | 41.4 | 38.8 | 95.1% | -0.09 |
| 21 | 2004 | nk | 7 | 343 | 1.63 | 268 | 8.7 | 155 | 99.2% | 10.5% | 411 | 43.8 | 39.5 | 95.3% | -0.16 |
| 22 | 2004 | nk | 8 | 608 | 1.68 | 475 | 10.7 | 98 | 99.3% | 8.0% | 436 | 28.5 | 26.4 | 81.8% | -0.18 |
| 23 | 2004 | nk | 12 | 444 | 1.63 | 347 | 9.8 | 167 | 99.0% | 14.8% | 410 | 45 | 41.4 | 95.5% | -0.01 |
| 24 | 2003 | nk | 10 | 824 | 1.78 | 500 | 8.8 | 134 | 99.3% | 7.3% | 457 | 39.4 | 38.5 | 97.6% | 0.02 |
| 25 | 2003 | nk | 12 | 780 | 1.8 | 500 | 8.3 | 134 | 99.3% | 7.8% | 456 | 39.1 | 37.6 | 97.5% | -0.05 |
| 26 | 2002 | nk | 10 | 846 | 1.72 | 500 | 5.8 | 149 | 99.3% | 8.4% | 447 | 43.3 | 40.8 | 97.7% | -0.04 |
| 27 | 2002 | nk | 12 | 871 | 1.75 | 500 | 8.7 | 149 | 99.3% | 8.7% | 445 | 42.9 | 40.7 | 97.8% | -0.01 |
| 28 | 2000 | nk | 8 | 793 | 1.86 | 500 | 7.0 | 126 | 99.3% | 8.4% | 449 | 36.6 | 35 | 94.6% | -0.04 |
| 29 | 2000 | nk | 12 | 1050 | 1.79 | 500 | 5.8 | 177 | 99.4% | 11.2% | 436 | 49.8 | 43.3 | 96.0% | -0.22 |
| **Avg.** |  |  | **10.1** | **645** | **1.74** | **439** | **8.7** | **135** | **99.3%** | **9.6%** | **421** | **38.6** | **36.4** | **91.6%** | **-0.07** |

**Supplementary Table 7: Additional quality metrics of aligned WGS reads from Illumina libraries prepared from California State Biobank DBS.** Abbreviations: OMIM: Mendelian inheritance in Man; CNV: copy number variant; CD: Coding Domain.

| **ID** | **Total Variants** | **Total SNVs** | **Total Indels** | **Mitoch-ondrial genome coverage** | **Total CD Variants** | **CNV calls overlap-ping CD of MIM genes** | **Mito-chondrial Variants** | **Average Genome Coverage** | **% Bases >Q30** | **Homo-zygous/ Hetero-zygous SNV Ratio** | **Trans-ition/ Trans-version Ratio** | **C>T+ G>A / T>C+ A>G variant ratio** | **Std. Dev./ Average Genome Coverage** | **Map-pable Genome (Gb)** |
| --- | --- | --- | --- | --- | --- | --- | --- | --- | --- | --- | --- | --- | --- | --- |
| 1 | 4,811,486 | 3,942,078 | 967,418 | 6,567 | 25,657 | 4 | 41 | 40 | 90.5 | 0.62 | 2.03 | 1.05 | 0.19 | 2.67 |
| 2 | 4,811,814 | 3,945,033 | 964,102 | 5,234 | 25,612 | 6 | 41 | 42 | 90.6 | 0.63 | 2.03 | 1.05 | 0.20 | 2.67 |
| 3 | 5,012,050 | 3,895,999 | 966,195 | 2,487 | 27,259 | 12 | 63 | 46 | 89.8 | 0.56 | 2.02 | 1.04 | 0.25 | 2.67 |
| 4 | 5,031,854 | 4,550,087 | 1,113,850 | 2,763 | 27,070 | 9 | 114 | 59 | 89.9 | 0.63 | 2.03 | 1.05 | 0.23 | 2.67 |
| 5 | 5,067,541 | 3,853,063 | 965,110 | 2,941 | 27,648 | 9 | 44 | 42 | 88.5 | 0.59 | 2.03 | 1.04 | 0.22 | 2.67 |
| 6 | 4,998,738 | 4,450,898 | 1,092,034 | 1,842 | 26,979 | 4 | 13 | 45 | 89.2 | 0.59 | 2.03 | 1.05 | 0.24 | 2.67 |
| 7 | 5,575,714 | 4,051,882 | 997,737 | 3,807 | 30,809 | 16 | 84 | 25 | 85.3 | 0.43 | 2.02 | 1.05 | 0.26 | 2.67 |
| 8 | 5,131,708 | 4,060,819 | 1,009,234 | 6,255 | 27,678 | 7 | 42 | 42 | 88.8 | 0.55 | 2.02 | 1.04 | 0.23 | 2.67 |
| 9 | 4,965,571 | 3,965,275 | 986,159 | 2,484 | 27,158 | 7 | 23 | 38 | 87.6 | 0.60 | 2.02 | 1.04 | 0.23 | 2.67 |
| 10 | 4,965,531 | 3,961,741 | 978,682 | 1,995 | 27,144 | 9 | 23 | 39 | 87.8 | 0.60 | 2.03 | 1.05 | 0.26 | 2.67 |
| 11 | 5,000,603 | 3,943,476 | 985,596 | 2,568 | 27,214 | 4 | 13 | 36 | 88.3 | 0.59 | 2.02 | 1.04 | 0.21 | 2.67 |
| 12 | 5,058,793 | 3,898,162 | 957,186 | 5,268 | 27,757 | 9 | 44 | 26 | 85.9 | 0.58 | 2.03 | 1.05 | 0.19 | 2.67 |
| 13 | 5,602,512 | 4,370,367 | 1,066,415 | 1,735 | 30,751 | 15 | 62 | 39 | 88.3 | 0.44 | 2.03 | 1.05 | 0.26 | 2.67 |
| 14 | 5,130,183 | 4,383,797 | 1,081,191 | 2,102 | 27,739 | 8 | 42 | 41 | 88.8 | 0.55 | 2.03 | 1.05 | 0.23 | 2.67 |
| 15 | 5,042,366 | 3,943,288 | 966,061 | 2,498 | 27,175 | 4 | 29 | 34 | 89.1 | 0.58 | 2.03 | 1.04 | 0.27 | 2.67 |
| 16 | 4,965,194 | 3,944,861 | 966,126 | 2,937 | 27,282 | 7 | 81 | 24 | 84.3 | 0.61 | 2.02 | 1.04 | 0.27 | 2.67 |
| 17 | 5,022,892 | 3,768,701 | 929,017 | 4,765 | 27,559 | 11 | 63 | 27 | 85.3 | 0.59 | 2.03 | 1.04 | 0.19 | 2.67 |
| 18 | 5,022,504 | 3,768,344 | 929,351 | 4,595 | 27,695 | 8 | 63 | 27 | 84.9 | 0.59 | 2.03 | 1.04 | 0.19 | 2.67 |
| 19 | 4,974,134 | 3,904,140 | 952,343 | 1,871 | 26,939 | 8 | 29 | 31 | 88.8 | 0.61 | 2.03 | 1.04 | 0.27 | 2.67 |
| 20 | 5,172,245 | 3,913,772 | 970,369 | 2,991 | 28,374 | 9 | 93 | 29 | 87.4 | 0.53 | 2.02 | 1.04 | 0.23 | 2.67 |
| 21 | 5,195,613 | 3,945,342 | 979,211 | 2,881 | 28,666 | 8 | 93 | 40 | 88.3 | 0.53 | 2.02 | 1.04 | 0.23 | 2.67 |
| 22 | 4,930,818 | 3,968,921 | 972,014 | 1,821 | 26,791 | 12 | 41 | 46 | 89.2 | 0.60 | 2.03 | 1.05 | 0.26 | 2.67 |
| 23 | 5,821,702 | 3,926,180 | 963,408 | 6,694 | 31,512 | 15 | 28 | 38 | 88.5 | 0.41 | 2.03 | 1.05 | 0.19 | 2.67 |
| 24 | 5,683,853 | 3,874,132 | 970,125 | 1,540 | 30,978 | 14 | 53 | 33 | 88.0 | 0.44 | 2.02 | 1.04 | 0.23 | 2.67 |
| 25 | 5,058,386 | 3,874,590 | 969,595 | 1,713 | 27,911 | 11 | 29 | 26 | 86.1 | 0.55 | 2.02 | 1.04 | 0.23 | 2.67 |
| 26 | 4,990,619 | 4,002,905 | 997,654 | 1,645 | 27,321 | 7 | 71 | 40 | 88.7 | 0.61 | 2.02 | 1.04 | 0.22 | 2.67 |
| 27 | 5,035,197 | 4,003,526 | 998,144 | 1,654 | 27,280 | 7 | 29 | 41 | 88.3 | 0.62 | 2.02 | 1.04 | 0.22 | 2.67 |
| 28 | 4,974,759 | 3,913,175 | 973,267 | 1,851 | 26,720 | 14 | 114 | 44 | 90.2 | 0.63 | 2.03 | 1.04 | 0.23 | 2.67 |
| 29 | 5,042,366 | 3,908,000 | 978,010 | 2,762 | 27,175 | 4 | 29 | 34 | 89.1 | 0.58 | 2.02 | 1.04 | 0.22 | 2.67 |
| **Avg.** | **5,106,784** | **3,997,674** | **987,779** | **3,113** | **27,788** | **9** | **52** | **37** | **88.1** | **0.57** | **2.03** | **1.04** | **0.23** | **2.67** |
| **SD** | **246,984** | **192,602** | **44,979** | **1,585** | **1,456** | **3.56** | **28.26** | **8.00** | **1.68** | **0.06** | **0.01** | **0.00** | **0.03** | **0.00** |

**Supplementary Table 8. Concordance analysis of single nucleotide variants (SNVs) and small insertions and deletions (indels) between WGS from EDTA blood and DBS.**

| **Sample ID** | **DBS type** | **Library Preparation Method** | **EDTA Blood Unique SNVs** | **DBS Sample Unique SNVs** | **SNVs Common to Blood and DBS** | **SNV Con-cordance** | **EDTA Blood Unique indels** | **DBS Sample Unique indels** | **indels Common to Blood and DBS** | **indel Con-cordance** |
| --- | --- | --- | --- | --- | --- | --- | --- | --- | --- | --- |
| 1 | FTA | Illumina | 6,634 | 12,514 | 3,041,847 | 99.4% | 4,783 | 7,356 | 492,376 | 97.6% |
| 1 | PC | Illumina | 6,604 | 12,942 | 3,041,883 | 99.4% | 5,146 | 7,517 | 492,001 | 97.5% |
| 1 | FTA | KAPA | 10,553 | 10,186 | 3,037,944 | 99.3% | 7,472 | 6,936 | 489,667 | 97.1% |
| 1 | PC | KAPA | 10,198 | 9,979 | 3,038,268 | 99.3% | 7,648 | 6,731 | 489,520 | 97.1% |
| 1 | PC | KAPA | 17,180 | 9,694 | 3,031,285 | 99.1% | 16,208 | 12,050 | 480,958 | 94.5% |
| 2 | FTA | KAPA | 8,016 | 11,056 | 3,085,526 | 99.4% | 6,285 | 9,108 | 500,429 | 97.0% |
| 2 | PC | KAPA | 10,681 | 10,062 | 3,082,849 | 99.3% | 8,844 | 9,514 | 497,875 | 96.4% |
| 2 | PC | KAPA | 11,154 | 12,799 | 3,082,394 | 99.2% | 7,852 | 9,973 | 498,858 | 96.6% |
| 3 | FTA | KAPA | 14,002 | 13,416 | 3,016,578 | 99.1% | 9,203 | 12,536 | 481,837 | 95.7% |
| 3 | PC | KAPA | 15,203 | 11,353 | 3,015,354 | 99.1% | 10,720 | 12,819 | 480,341 | 95.3% |
| 3 | PC | KAPA | 15,718 | 13,911 | 3,014,836 | 99.0% | 14,005 | 15,152 | 477,055 | 94.2% |
| 4 | FTA | Illumina | 6,608 | 14,026 | 3,029,084 | 99.3% | 5,548 | 8,487 | 489,090 | 97.2% |
| 4 | PC | Illumina | 6,602 | 13,471 | 3,029,090 | 99.3% | 5,229 | 8,281 | 489,405 | 97.3% |
| 4 | FTA | KAPA | 9,172 | 11,351 | 3,026,531 | 99.3% | 6,724 | 8,428 | 487,897 | 97.0% |
| 4 | PC | KAPA | 17,734 | 11,412 | 3,017,943 | 99.0% | 13,233 | 11,790 | 481,408 | 95.1% |
| 5 | FTA | KAPA | 9,350 | 10,497 | 3,025,747 | 99.3% | 7,170 | 7,912 | 488,936 | 97.0% |
| 5 | PC | KAPA | 14,253 | 9,429 | 3,020,826 | 99.2% | 12,816 | 10,299 | 483,308 | 95.4% |
| 5 | PC | KAPA | 15,255 | 8,328 | 3,019,829 | 99.2% | 11,522 | 8,381 | 484,594 | 96.1% |
| 6 | FTA | KAPA | 11,605 | 11,430 | 3,023,977 | 99.2% | 7,768 | 9,358 | 486,238 | 96.6% |
| 6 | PC | KAPA | 18,507 | 11,747 | 3,017,078 | 99.0% | 17,337 | 13,723 | 476,666 | 93.9% |
| 6 | PC | KAPA | 16,898 | 12,726 | 3,018,686 | 99.0% | 12,606 | 11,888 | 481,393 | 95.2% |
| 7 | FTA | KAPA | 11,706 | 12,131 | 3,018,391 | 99.2% | 8,196 | 9,061 | 486,761 | 96.6% |
| 7 | PC | KAPA | 11,982 | 9,901 | 3,018,102 | 99.3% | 9,625 | 7,747 | 485,347 | 96.5% |
| 7 | PC | KAPA | 11,545 | 11,925 | 3,018,549 | 99.2% | 7,153 | 7,457 | 487,808 | 97.1% |
| 8 | FTA | KAPA | 12,171 | 12,580 | 3,456,999 | 99.3% | 8,553 | 9,155 | 561,592 | 96.9% |
| 8 | PC | KAPA | 19,201 | 10,009 | 3,449,945 | 99.2% | 10,866 | 10,124 | 559,300 | 96.4% |
| 9 | FTA | KAPA | 9,668 | 11,421 | 3,036,003 | 99.3% | 7,621 | 8,170 | 493,979 | 96.9% |
| 9 | PC | KAPA | 12,205 | 9,266 | 3,033,444 | 99.3% | 11,382 | 8,782 | 490,227 | 96.0% |
| 10 | FTA | Illumina | 12,423 | 17,305 | 3,682,496 | 99.2% | 15,456 | 15,131 | 594,647 | 95.1% |
| 10 | PC | Illumina | 9,930 | 19,230 | 3,684,996 | 99.2% | 9,013 | 13,653 | 601,095 | 96.4% |
| 10 | FTA | KAPA | 13,685 | 15,381 | 3,681,259 | 99.2% | 10,135 | 12,826 | 599,951 | 96.3% |
| 10 | PC | KAPA | 17,205 | 19,931 | 3,677,745 | 99.0% | 13,125 | 18,942 | 596,933 | 94.9% |
| 10 | PC | KAPA | 24,960 | 17,672 | 3,669,986 | 98.9% | 15,722 | 15,513 | 594,348 | 95.0% |
| 11 | FTA | Illumina | 7,263 | 12,578 | 3,032,422 | 99.3% | 4,653 | 7,046 | 491,035 | 97.7% |
| 11 | FTA | Illumina | 7,634 | 11,911 | 3,032,051 | 99.4% | 5,250 | 7,083 | 490,432 | 97.5% |
| 11 | PC | Illumina | 7,606 | 12,253 | 3,032,083 | 99.3% | 5,361 | 7,053 | 490,312 | 97.5% |
| 11 | PC | KAPA | 13,320 | 9,771 | 3,026,363 | 99.2% | 11,483 | 9,260 | 484,189 | 95.9% |
| 11 | PC | KAPA | 10,029 | 7,838 | 3,029,644 | 99.4% | 6,774 | 5,752 | 488,914 | 97.5% |
| 12 | FTA | KAPA | 19,286 | 11,840 | 3,054,440 | 99.0% | 10,642 | 10,027 | 489,312 | 95.9% |
| 12 | PC | KAPA | 11,758 | 11,809 | 3,061,928 | 99.2% | 10,633 | 10,745 | 489,352 | 95.8% |
| 12 | PC | KAPA | 11,250 | 10,723 | 3,062,431 | 99.3% | 8,289 | 7,456 | 491,701 | 96.9% |
| 13 | FTA | Illumina | 6,939 | 13,588 | 3,140,921 | 99.4% | 5,205 | 7,945 | 509,347 | 97.5% |
| 13 | FTA | Illumina | 7,281 | 14,130 | 3,140,589 | 99.3% | 6,490 | 8,415 | 508,048 | 97.1% |
| 13 | PC | Illumina | 6,661 | 13,913 | 3,141,203 | 99.3% | 5,211 | 7,894 | 509,342 | 97.5% |
| 13 | PC | KAPA | 14,747 | 10,001 | 3,133,111 | 99.2% | 11,227 | 8,275 | 503,324 | 96.3% |
| 14 | FTA | Illumina | 5,721 | 14,666 | 3,060,376 | 99.3% | 4,936 | 6,990 | 497,840 | 97.7% |
| 14 | PC | Illumina | 5,882 | 14,855 | 3,060,207 | 99.3% | 5,311 | 7,091 | 497,473 | 97.6% |
| 14 | PC | KAPA | 8,754 | 9,223 | 3,057,335 | 99.4% | 7,452 | 5,742 | 495,335 | 97.4% |
| 15 | FTA | KAPA | 20,004 | 9,529 | 3,005,792 | 99.0% | 10,573 | 9,175 | 481,854 | 96.1% |
| 15 | PC | KAPA | 16,065 | 9,372 | 3,009,736 | 99.2% | 12,167 | 10,865 | 480,262 | 95.4% |
| 16 | PC | KAPA | 12,941 | 9,208 | 3,006,604 | 99.3% | 6,909 | 8,158 | 483,157 | 97.0% |
| 17 | PC | KAPA | 21,124 | 8,785 | 3,042,819 | 99.0% | 8,869 | 7,140 | 490,338 | 96.8% |
| 18 | FTA | Illumina | 6,575 | 12,035 | 3,026,843 | 99.4% | 7,060 | 9,603 | 484,887 | 96.7% |
| 18 | PC | Illumina | 6,669 | 12,081 | 3,026,750 | 99.4% | 7,521 | 9,621 | 484,420 | 96.6% |
| 19 | FTA | Illumina | 6,308 | 12,797 | 3,018,212 | 99.4% | 7,498 | 10,541 | 483,581 | 96.4% |
| 19 | PC | Illumina | 7,267 | 12,589 | 3,017,251 | 99.3% | 10,097 | 11,305 | 480,977 | 95.7% |
| 20 | FTA | Illumina | 9,123 | 13,756 | 3,039,086 | 99.3% | 5,686 | 9,308 | 490,093 | 97.0% |
| 21 | FTA | Illumina | 9,055 | 15,059 | 3,018,481 | 99.2% | 6,522 | 11,797 | 484,642 | 96.4% |
| 22 | FTA | Illumina | 8,734 | 13,506 | 3,020,837 | 99.3% | 5,178 | 7,724 | 487,306 | 97.4% |
| 23 | FTA | Illumina | 6,892 | 15,744 | 3,059,750 | 99.3% | 6,134 | 11,086 | 493,958 | 96.6% |
| 24 | FTA | Illumina | 9,543 | 17,335 | 3,049,393 | 99.1% | 7,539 | 13,165 | 488,270 | 95.9% |
| 25 | FTA | Illumina | 7,844 | 14,284 | 3,031,351 | 99.3% | 5,393 | 9,119 | 490,201 | 97.1% |

**Supplementary Figure**

**Supplementary Figure 1: Impact of DBS variables on WGS quality. a.** Comparison of age of DBS with genome coverage by WGS of DNA derived from those DBS. **b.** Comparison of age of DBS with average coverage of Mendelian Inheritance in Man genes by WGS of DNA derived from those DBS. **c.** Comparison of age of DBS with raw WGS yield of DNA derived from those DBS. **d.** Comparison of genome coverage by WGS with DBS filter paper type used for California newborn screening. Error bars are standard deviation.

**
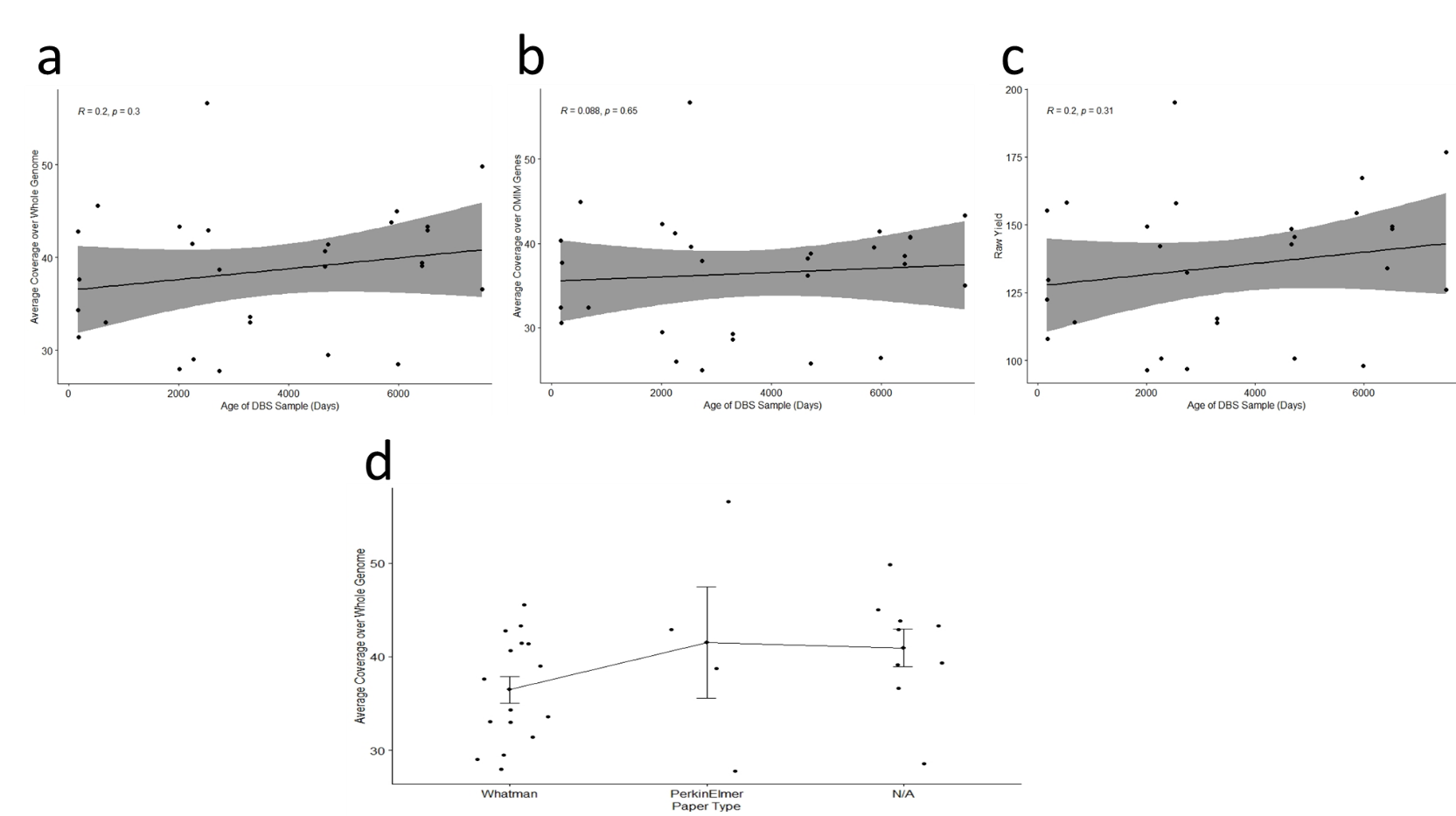
**

**Supplementary Figure 2:** Chr 1:187,677,560-187,677,598, an AT-rich, non-coding region showing a heterozygous dinucleotide deletion and a discordant, overlapping heterozygous T>A substitution. Shown, from top to bottom, are the reference nucleotide sequence, average coverage in WGS from 200 unrelated subjects, and coverage and representative reads from a proband (blood sample), father (blood sample), sibling (blood sample), father (DBS, KAPA library) father (DBS, Illumina library), proband (DBS, KAPA library), proband (DBS, Illumina library), and RepeatMasker.


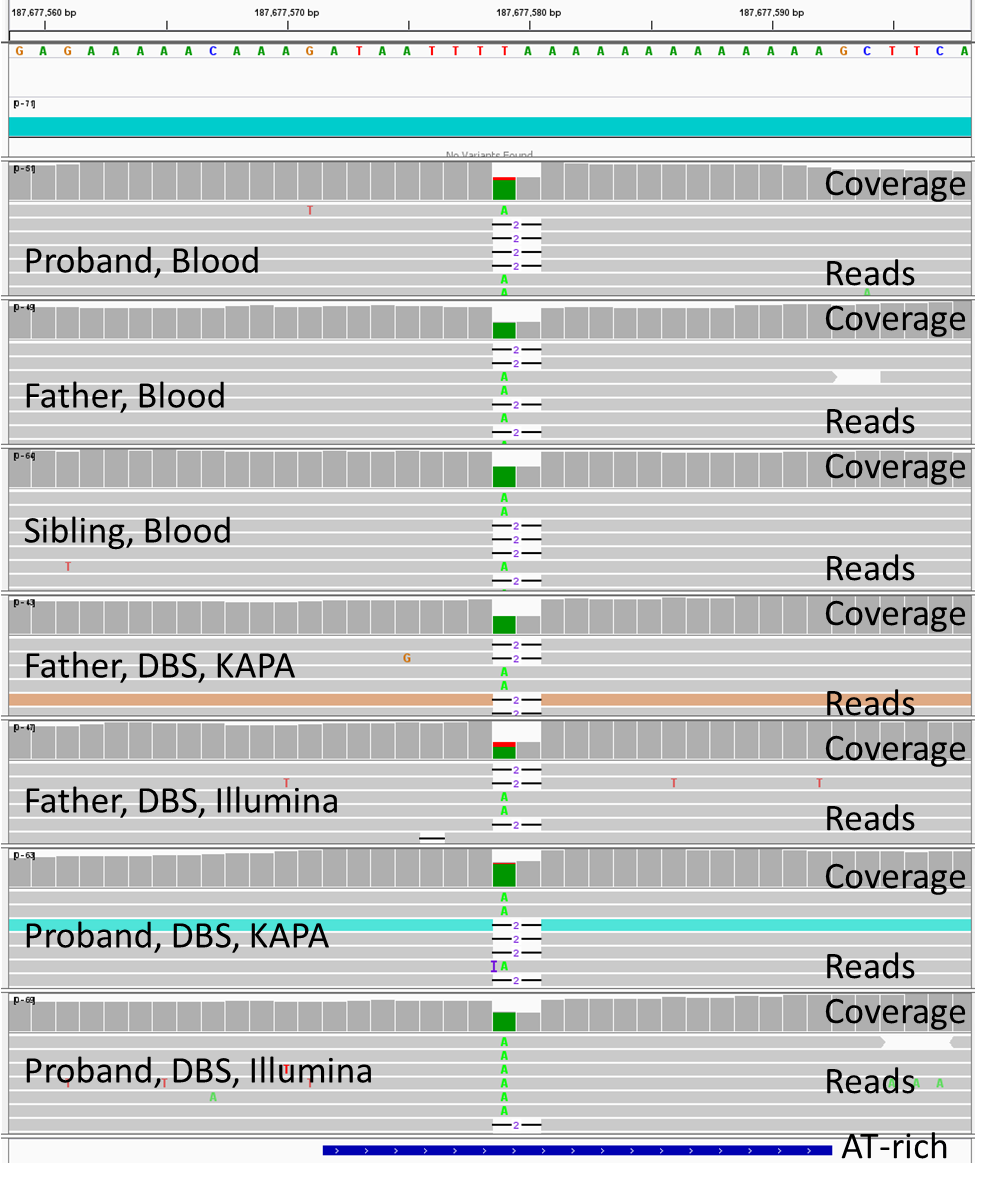


**Supplementary Figure 3:** Chr 18:21,542,872-21,542,910, a non-coding region featuring an Alu element, showing a heterozygous tetranucleotide deletion and a discordant, overlapping heterozygous A>G substitution. Shown, from top to bottom, are the reference nucleotide sequence, average coverage in WGS from 200 unrelated subjects, and coverage and representative reads from a proband (blood sample), father (blood sample), sibling (blood sample), father (DBS, KAPA library) father (DBS, Illumina library), proband (DBS, KAPA library), proband (DBS, Illumina library), and RepeatMasker.


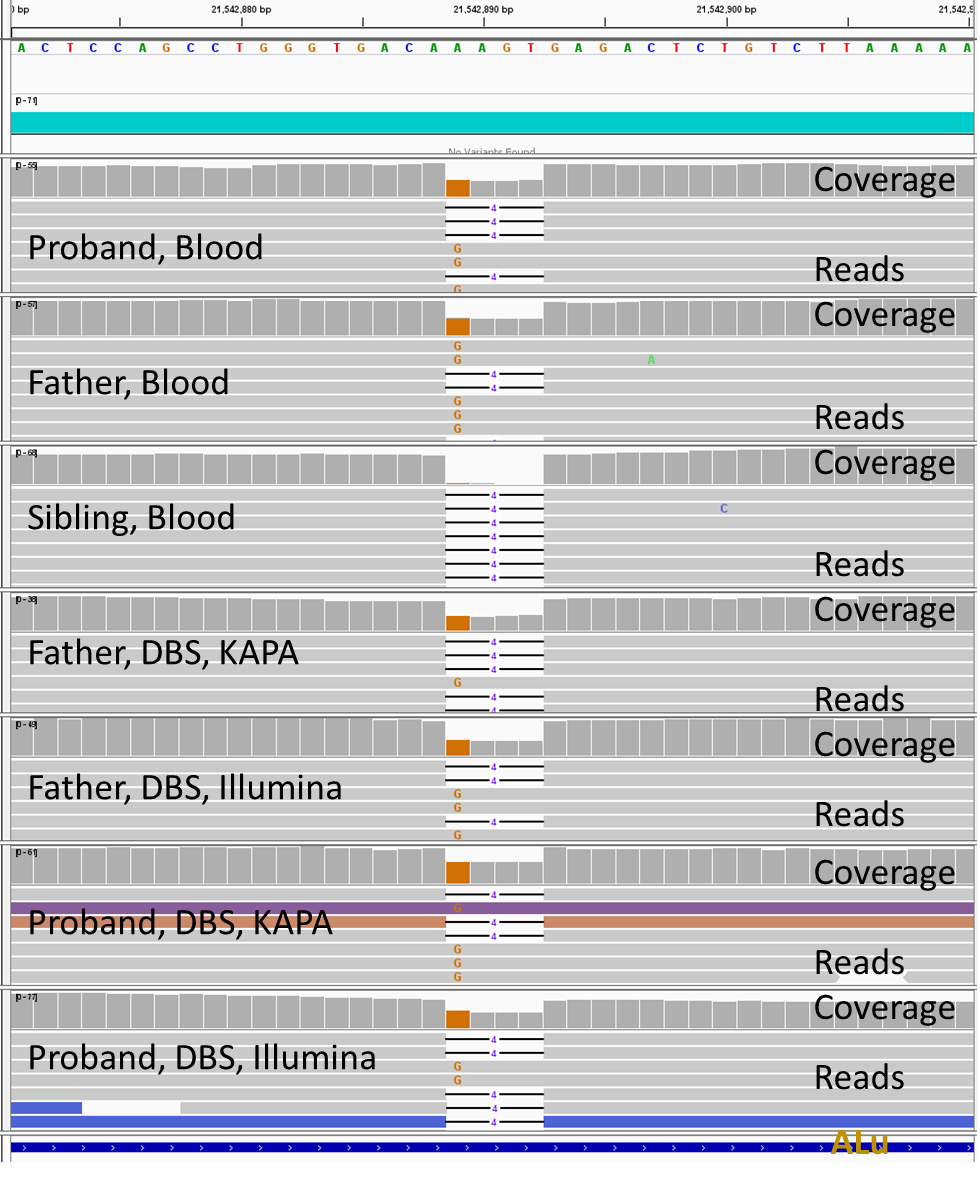
**Supplementary Figure 4:** Chr 4:171,247,140-171,247,178, an AT-rich, non-coding region showing a heterozygous, single nucleotide deletion with a discordant, overlapping heterozygous T>A substitution in some samples. The sibling is homozygous for the deletion. Shown, from top to bottom, are the reference nucleotide sequence, average coverage in WGS from 200 unrelated subjects, and coverage and representative reads from a proband (blood sample), father (blood sample), sibling (blood sample), father (DBS, KAPA library) father (DBS, Illumina library), proband (DBS, KAPA library), proband (DBS, Illumina library), and RepeatMasker.


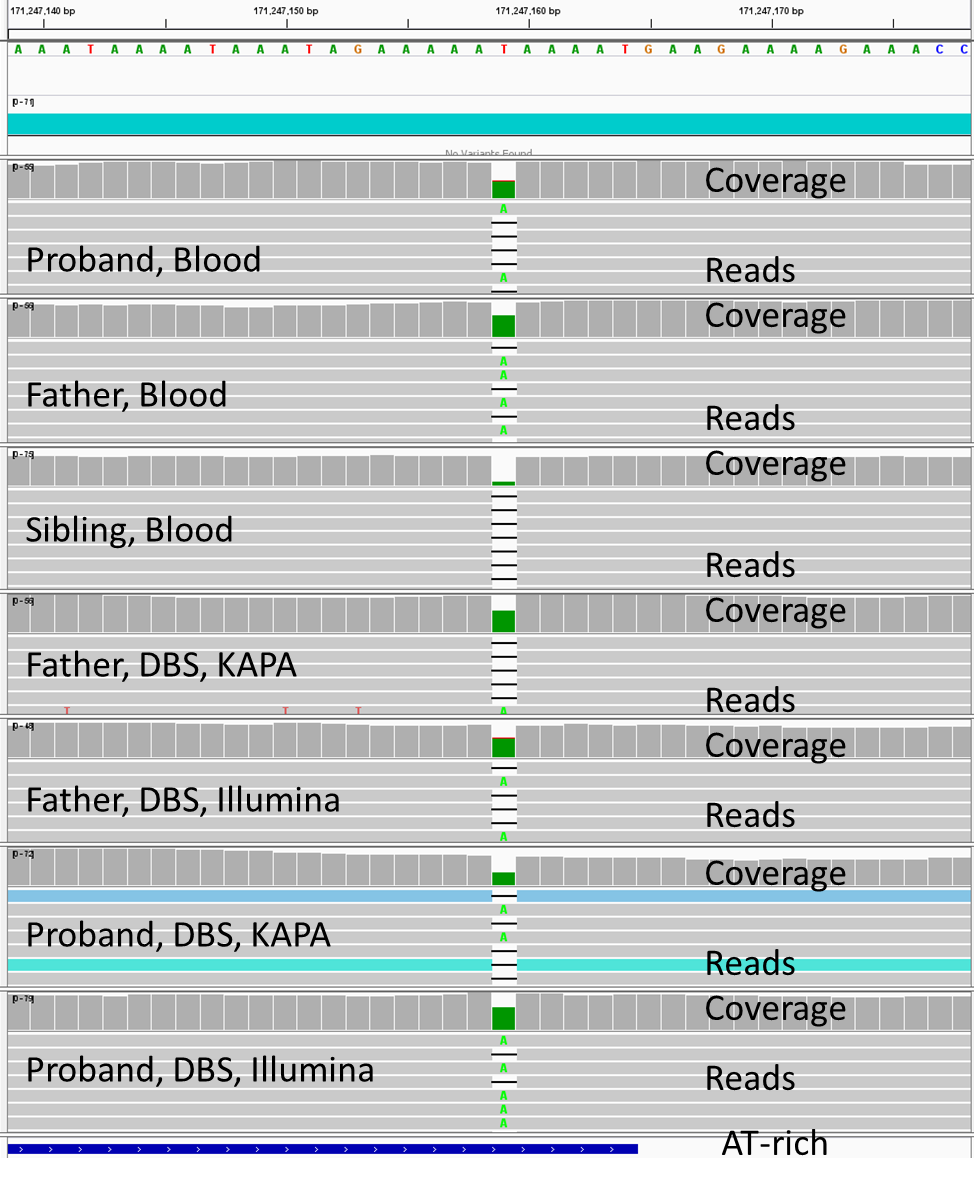


**Supplementary Figure 5:** Chr 3:5,211,240-5,211,271, a CT-rich region of a LINE1 retrotransposon in an intron of ARL8B showing a heterozygous, single nucleotide deletion with a discordant, overlapping heterozygous C>T substitution in in the father. Shown, from top to bottom, are the reference nucleotide sequence, gene, average WGS coverage of 200 unrelated subjects, and coverage and representative reads from a proband (blood sample), father (blood sample), sibling (blood sample), father (DBS, KAPA library) father (DBS, Illumina library), proband (DBS, KAPA library), proband (DBS, Illumina library), and RepeatMasker.


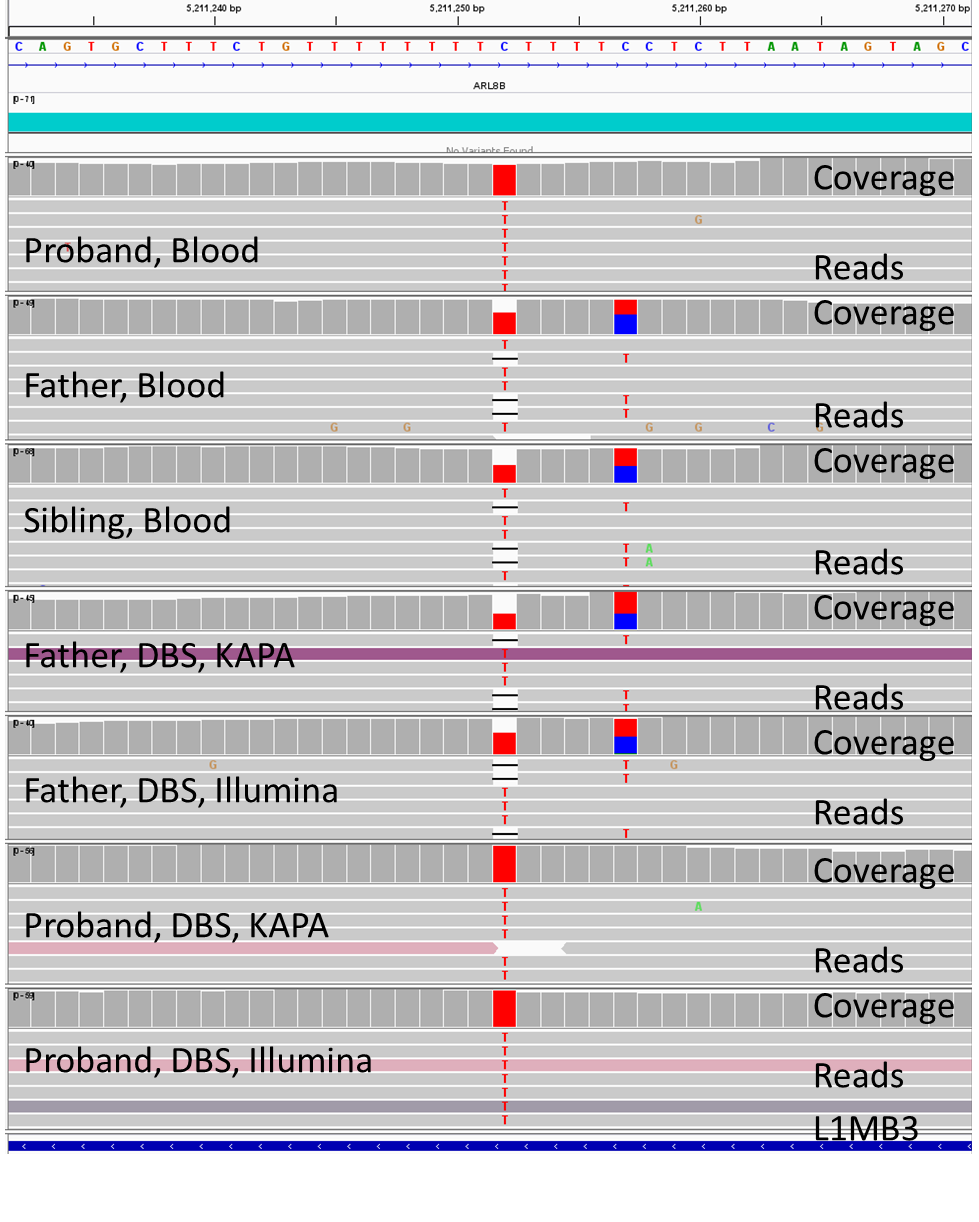


**Supplementary Figure 6:** Chr 10:132,112,292-132,112,330, a non-coding, TTA-repeat, LINE1-containing region showing 3 discrepant, overlapping variants: A homozygous or heterozygous trinucleotide deletion overlapping with a heterozygous A>T substitution which was also called as a TA>T single nucleotide deletion. This may be a random alignment error. Shown, from top to bottom, are the reference nucleotide sequence, average WGS coverage in 200 unrelated subjects, and coverage and representative reads from a proband (blood sample), father (blood sample), sibling (blood sample), father (DBS, KAPA library) father (DBS, Illumina library), proband (DBS, KAPA library), proband (DBS, Illumina library), and RepeatMasker.


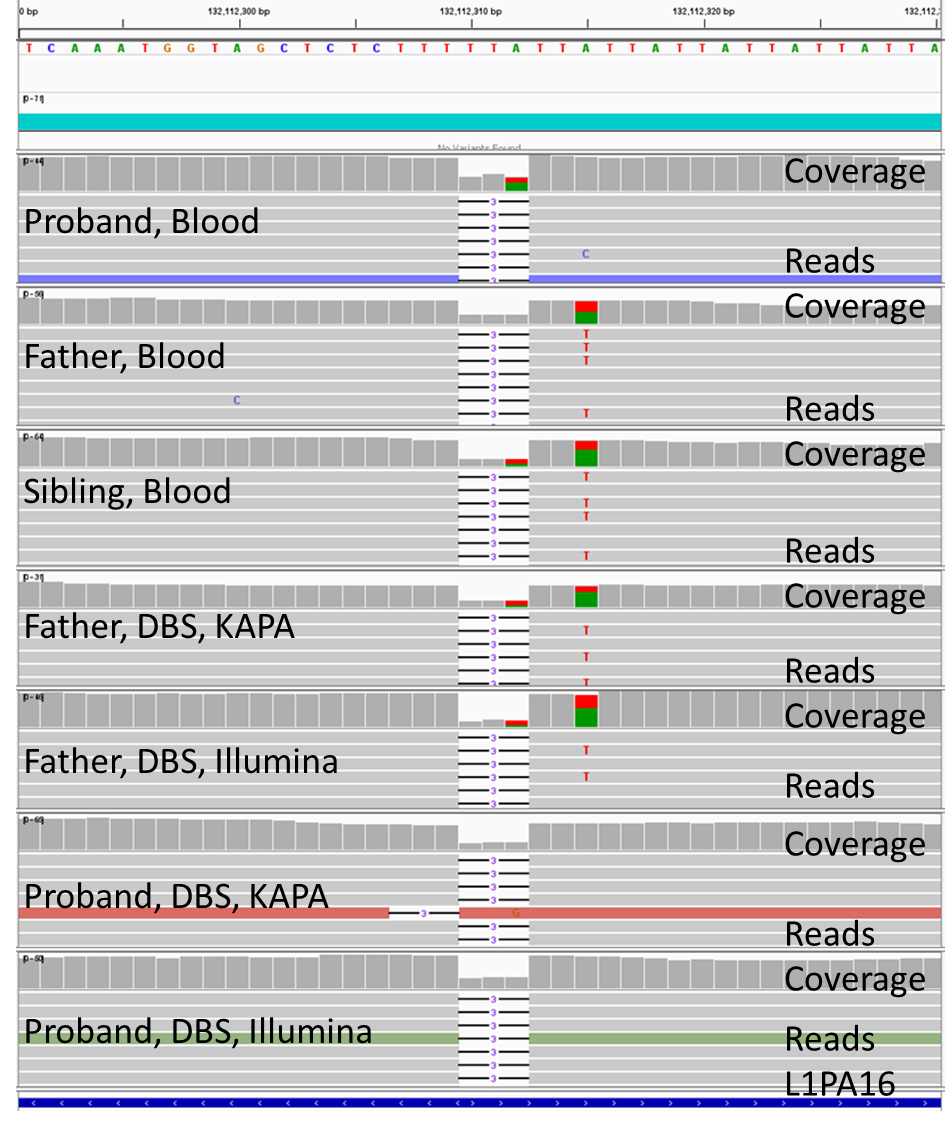


**Supplementary Figure 7:** Chr 13:64,108,481-64,108,519, a non-coding LINE1 repeat-containing region showing a discrepant 12-nucleotide homozygous or heterozygous deletion based on depth of coverage for the two alleles. Shown, from top to bottom, are the reference nucleotide sequence, average WGS coverage in 200 unrelated subjects, and coverage and representative reads from a proband (blood sample), father (blood sample), sibling (blood sample), father (DBS, KAPA library) father (DBS, Illumina library), proband (DBS, KAPA library), proband (DBS, Illumina library), and RepeatMasker.


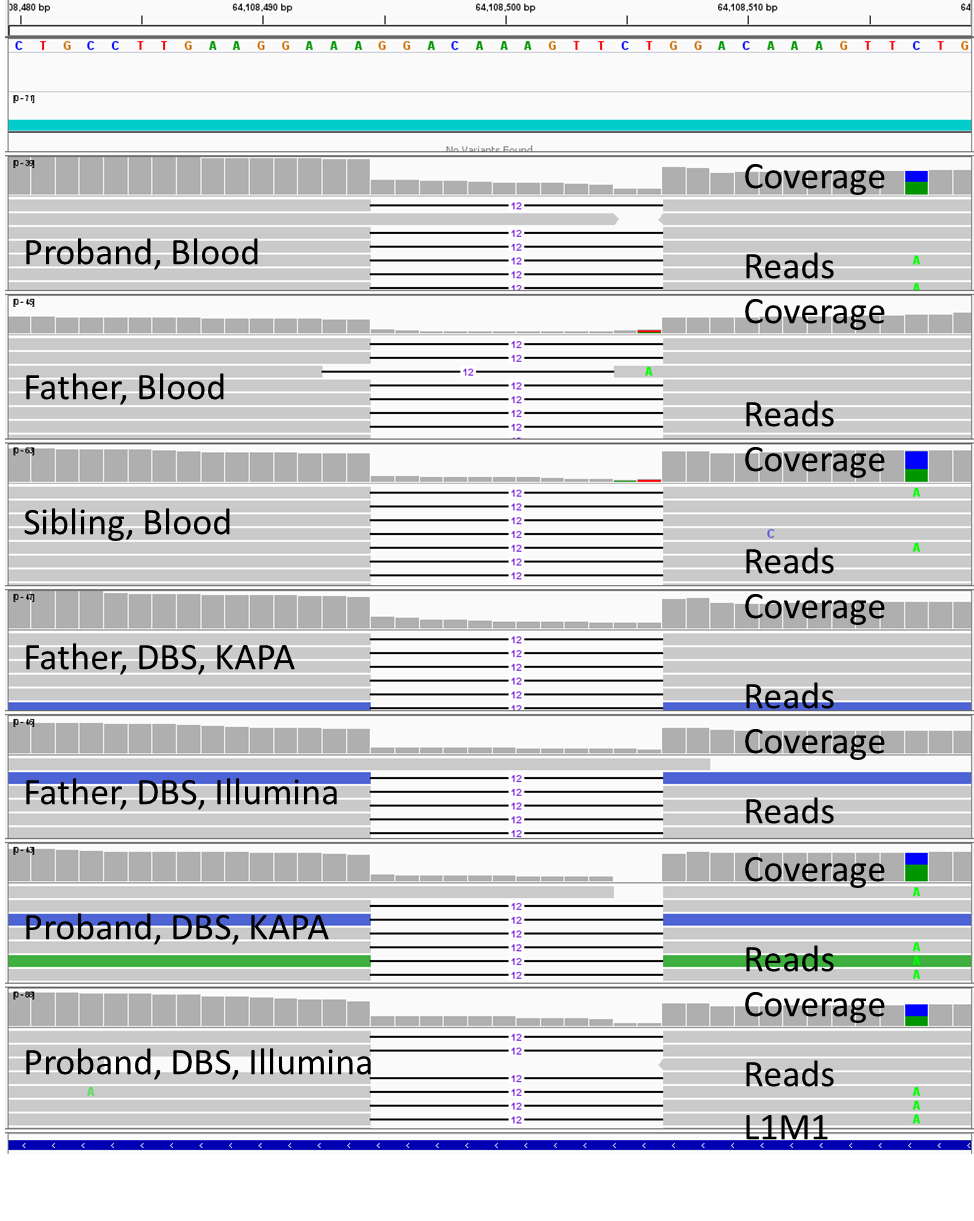


**Supplementary Figure 8:** Chr 4:171,247,140-171,247,178, a non-coding, AT-rich region, showing a discrepant overlapping homozygous or heterozygous T>A substitution and heterozygous single nucleotide deletion. Shown, from top to bottom, are the reference nucleotide sequence, average WGS coverage in 200 unrelated subjects, and coverage and representative reads from a proband (blood sample), father (blood sample), sibling (blood sample), father (DBS, KAPA library) father (DBS, Illumina library), proband (DBS, KAPA library), proband (DBS, Illumina library), and RepeatMasker.


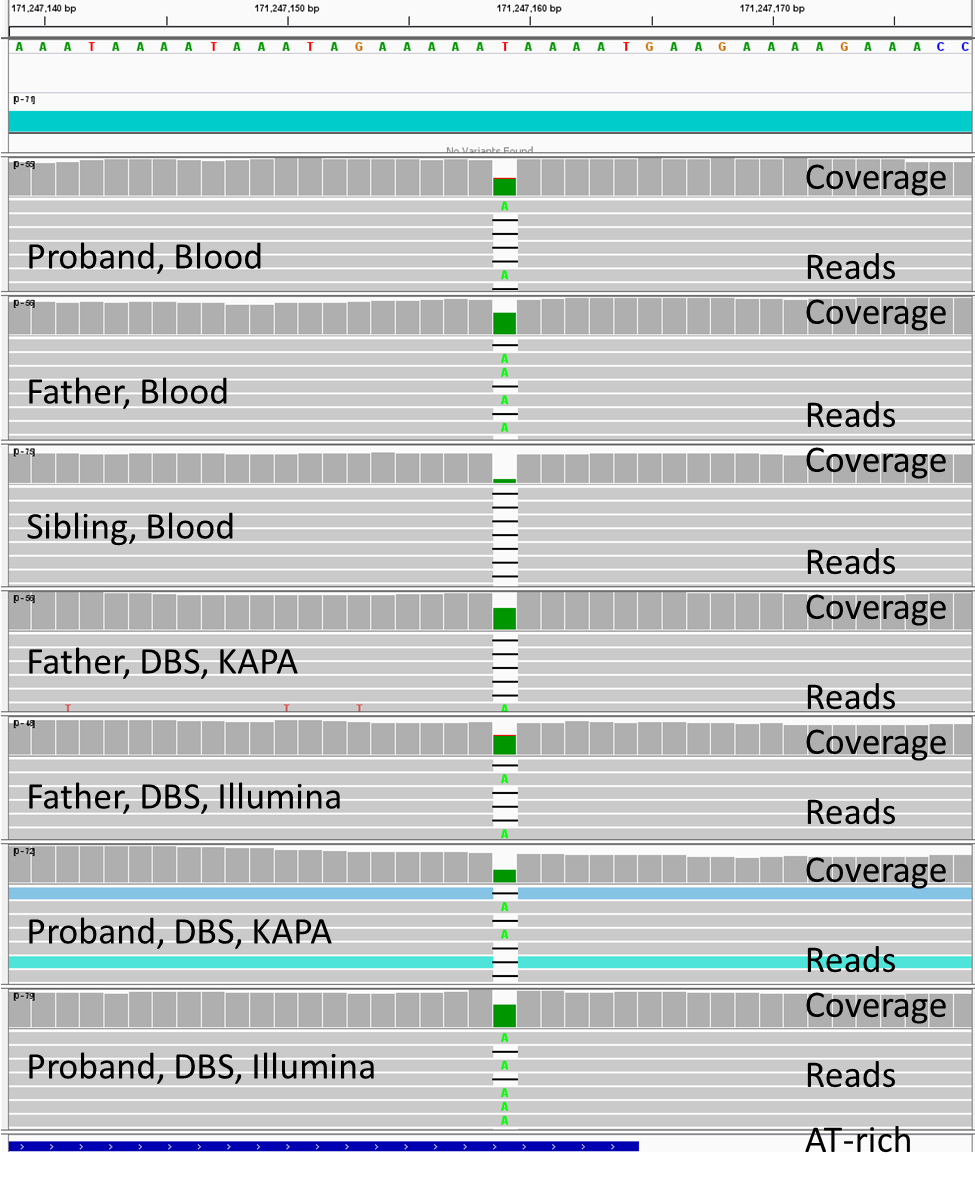


**Supplementary Figure 9:** Chr 7:67,120,947-67,121,106, a region containing an MSTB1 long terminal repeat endogenous retrovirus, showing a discrepant homozygous or heterozygous C>A substitution. Shown, from top to bottom, are the reference nucleotide sequence, average WGS coverage in 200 unrelated subjects, and coverage and representative reads from a proband (blood sample), father (blood sample), sibling (blood sample), father (DBS, KAPA library) father (DBS, Illumina library), proband (DBS, KAPA library), proband (DBS, Illumina library), and RepeatMasker.


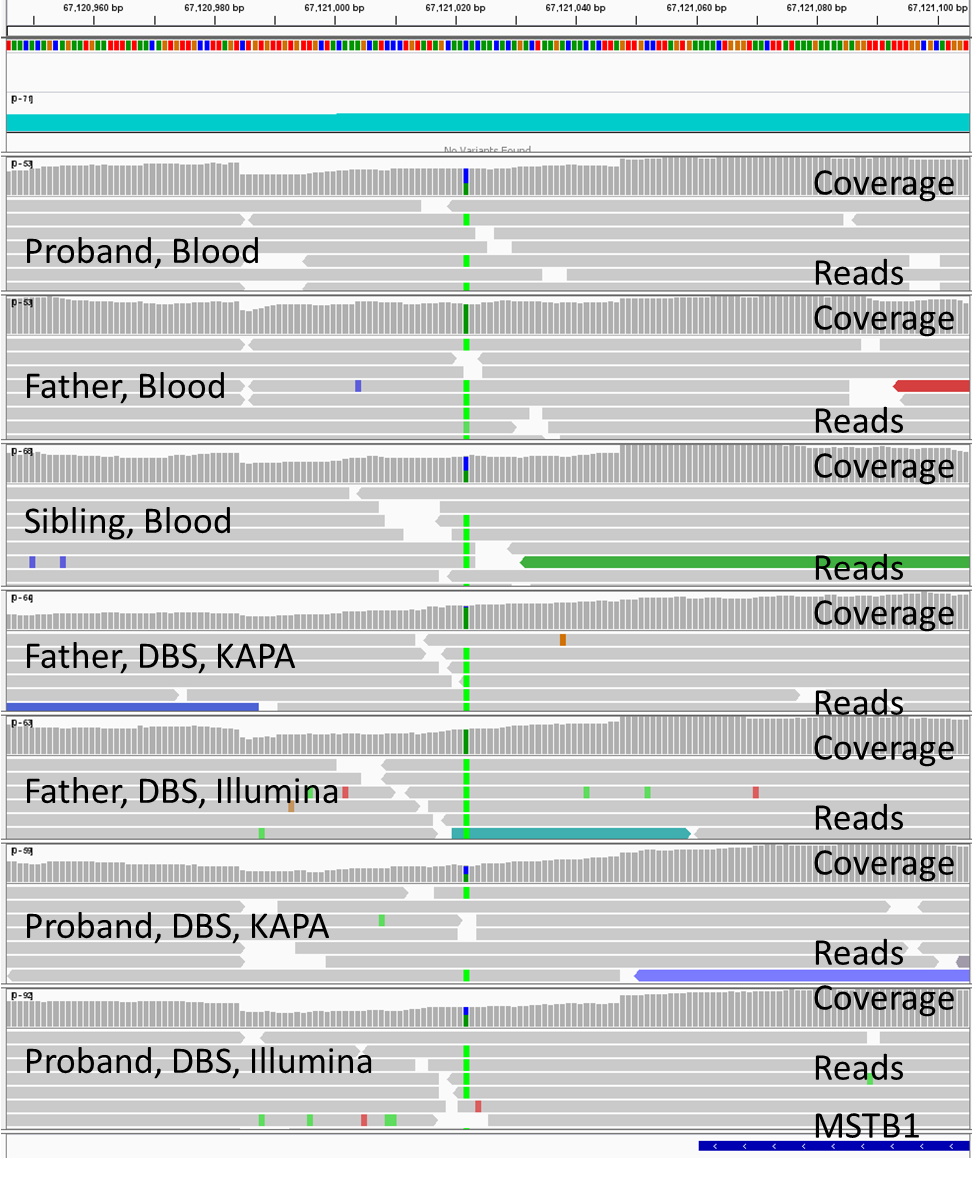


**Supplementary Figure 10**: Chr 10:133,159,143-133,159,981, showing a non-coding region that contains a discrepant single versus two nucleotide deletion in a 9 nucleotide guanine homopolymer. Shown, from top to bottom, are the reference nucleotide sequence, average WGS coverage in 200 unrelated subjects, and coverage and representative reads from a proband (blood sample), father (blood sample), sibling (blood sample), father (DBS, KAPA library) father (DBS, Illumina library), proband (DBS, KAPA library), proband (DBS, Illumina library), and RepeatMasker.
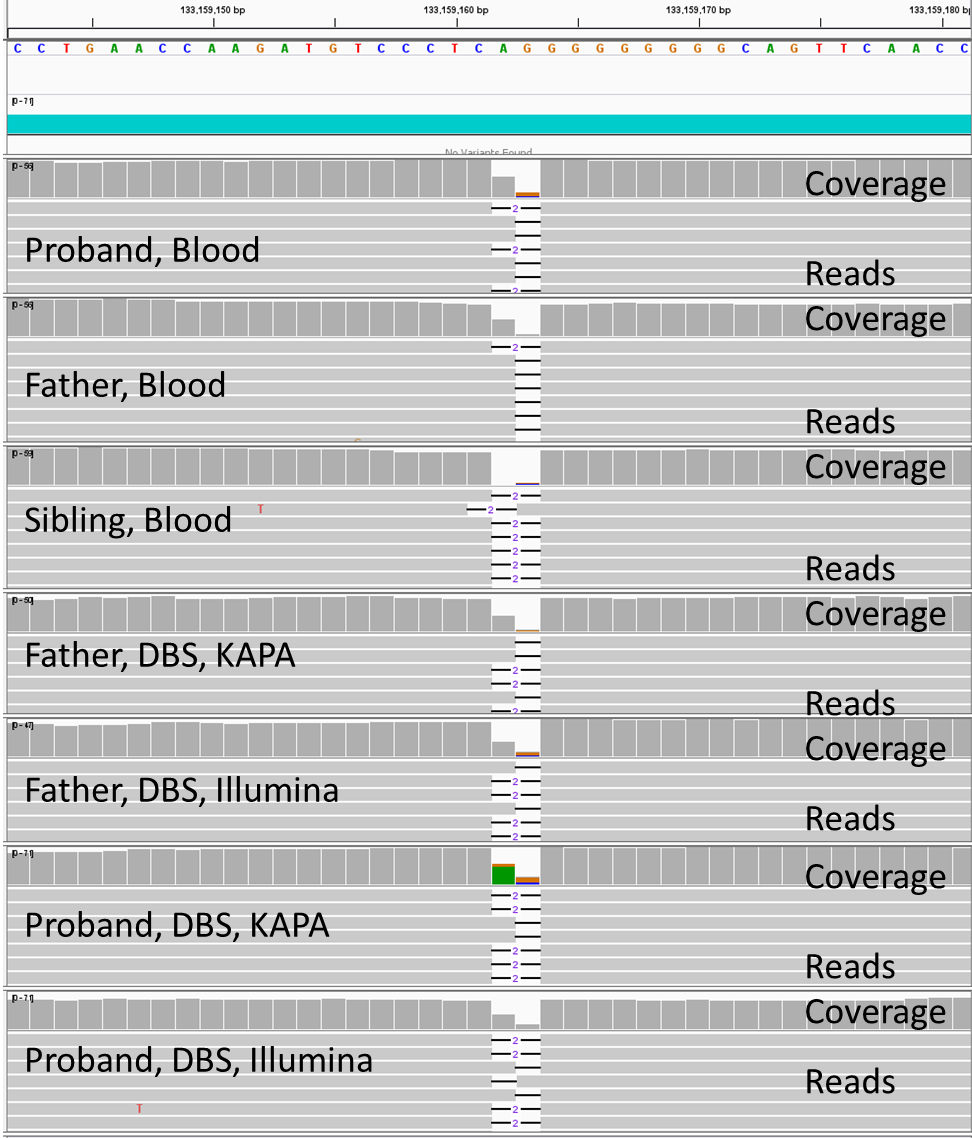


**Supplementary Figure 11:** Chr 11: 110,204,648-110,204,686 showing a non-coding region that contains an Alu repetitive element, a heterozygous 5 nucleotide deletion within a polythymidine tract, and a discordant, overlapping, heterozygous single nucleotide insertion.


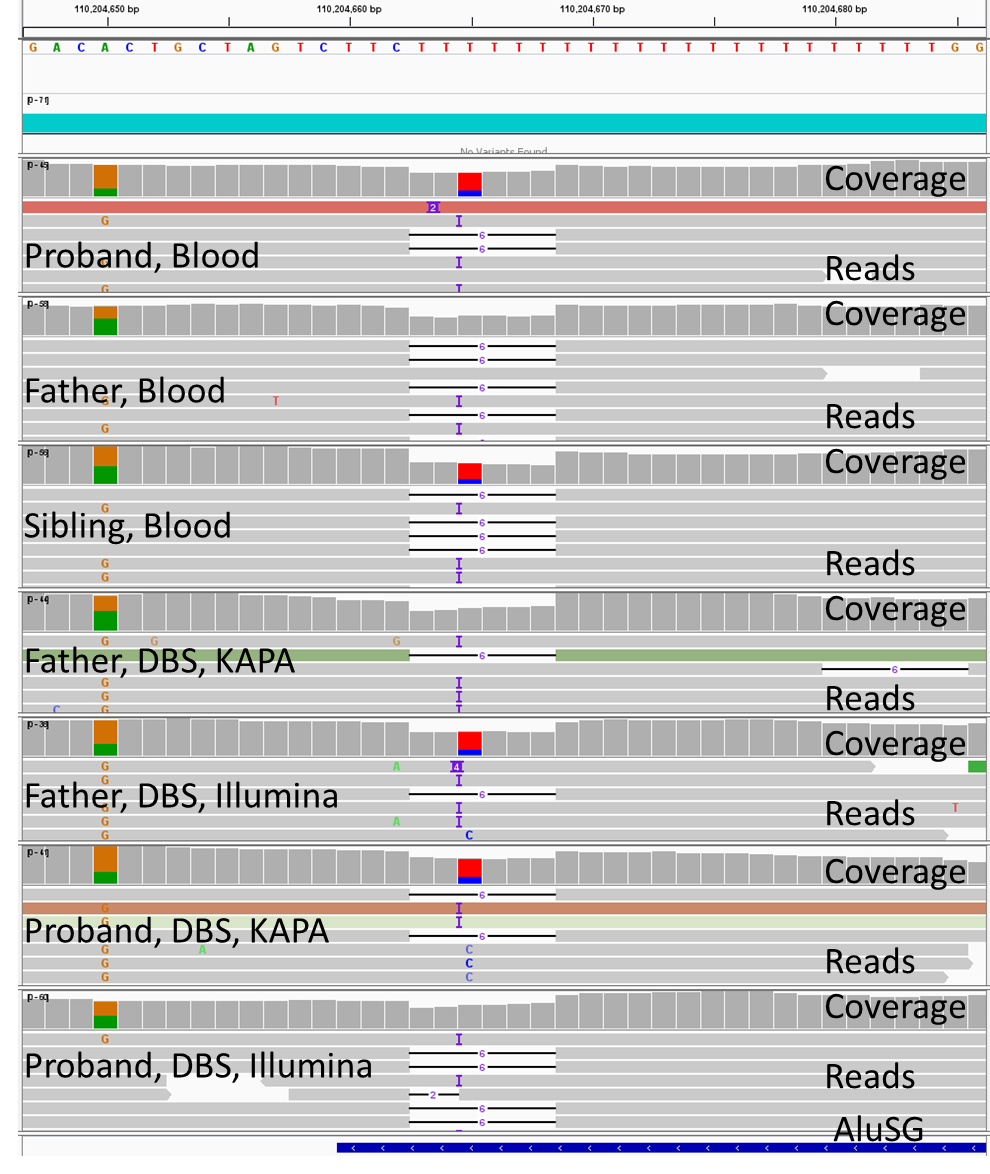


**Supplementary Figure 12:** Chr 4:67,030,778-67,030,816, a non-coding polythymidine tract within a MER repetitive element showing a discordant heterozygous A>T substitution overlapping a thymidine mononucleotide deletion.


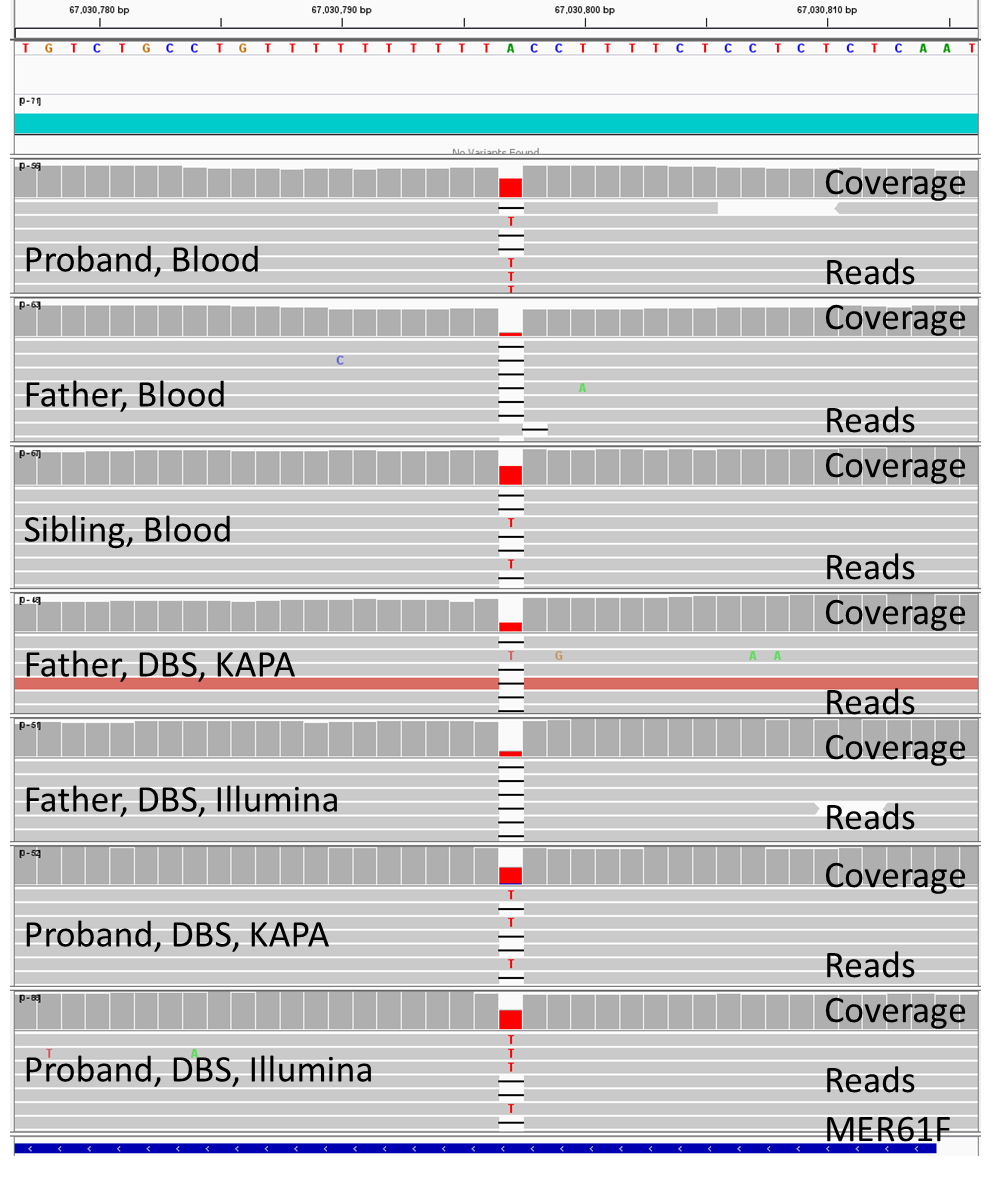


**Supplementary Figure 13:** Chr 2: 213,184,938-213,184,976 showing an intron of *ERBB4* containing 4 variants within a polythymidine tract. They are a heterozygous haplotype comprising a single nucleotide insertion and an A>T substitution, a single nucleotide deletion overlapping the A>T substitution (which was called homozygous or heterozygous in different sample types), and a haplotype comprising the A>T substitution (without the insertion) and a C>T substitution.


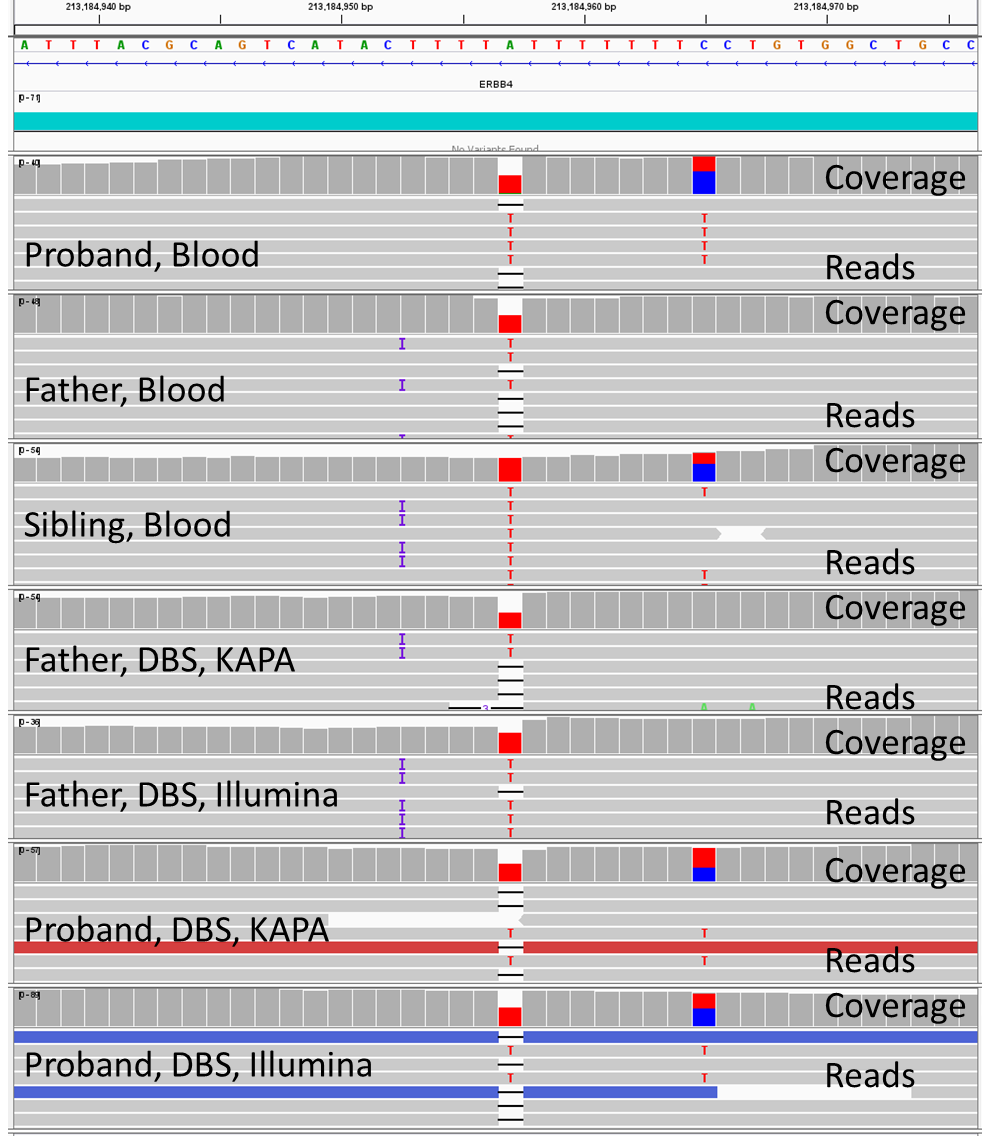


**Supplementary Figure 14:** Chr. 14:78,838,500-78,838,538 showing a non-coding pentathymidine tract adjacent to a 9 adenine homopolymer containing a discrepant homozygous or heterozygous thymidine deletion and T>A substitution together with an overlapping heterozygous TT>AA substitution.


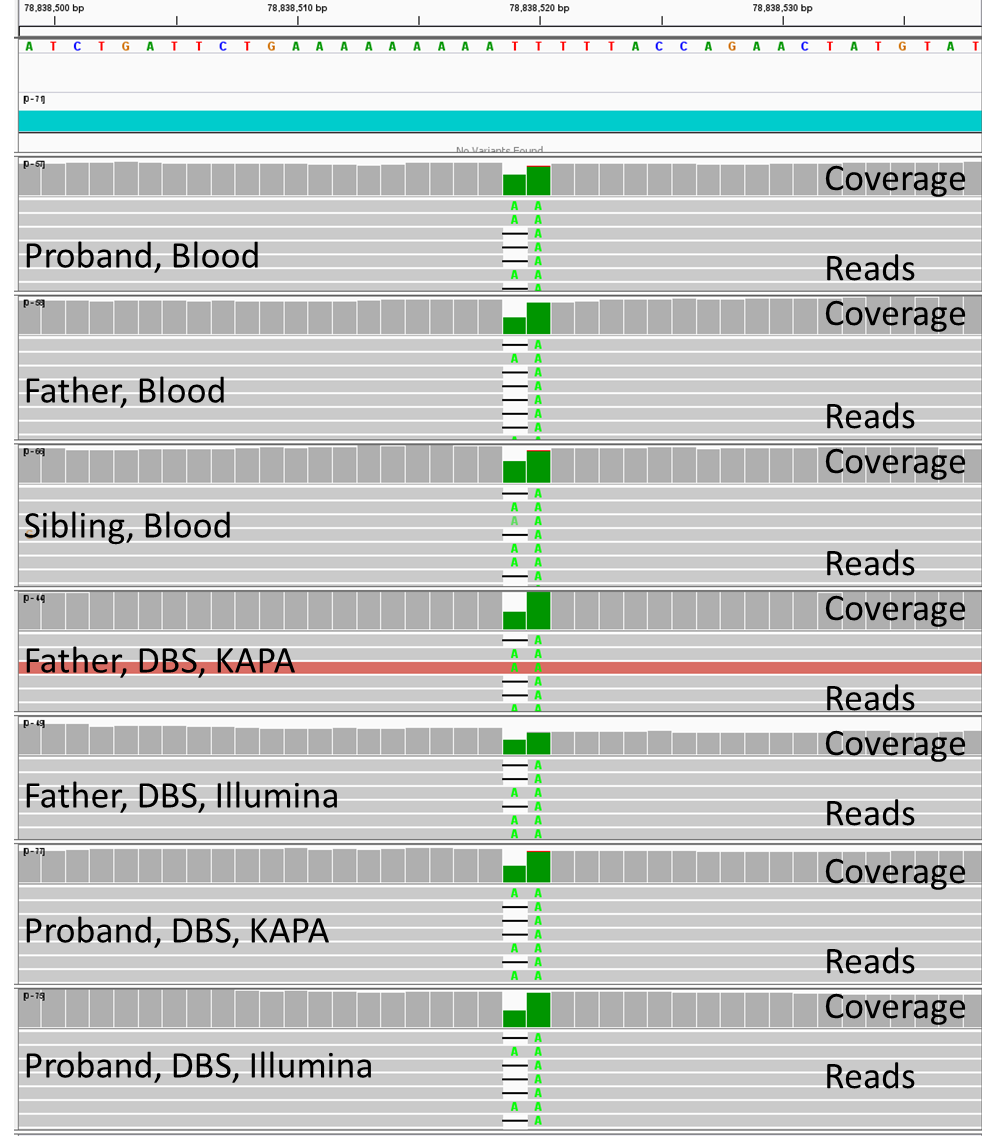


**Supplementary Figure 15:** Chr 11:107,991,604-107,991,642 showing a non-coding region with a FLAM element flanked by a homoadenine tract with a discrepant heterozygous versus homozygous A>G substitution and 1-base deletion haplotype in the proband, and an overlapping, discordant, heterozygous versus homozygous A>G substitution in the father.


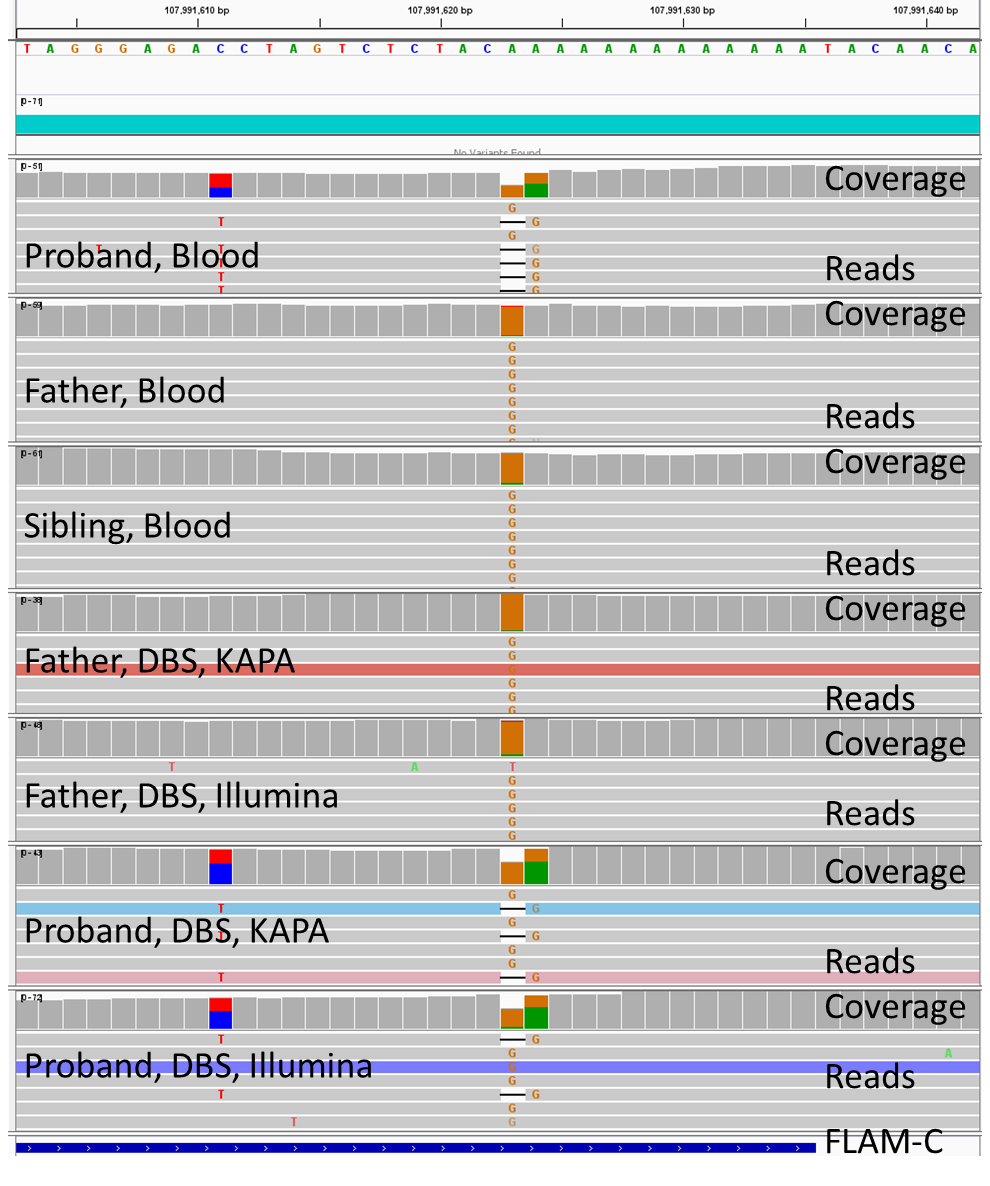


**Supplementary Figure 16:** Chr 7:3,185,475-3,185,513, a non-coding region containing an Alu repeat with a discordant heterozygous versus homozygous single nucleotide deletion adjacent to a heterozygous versus homozygous C>T substitution.


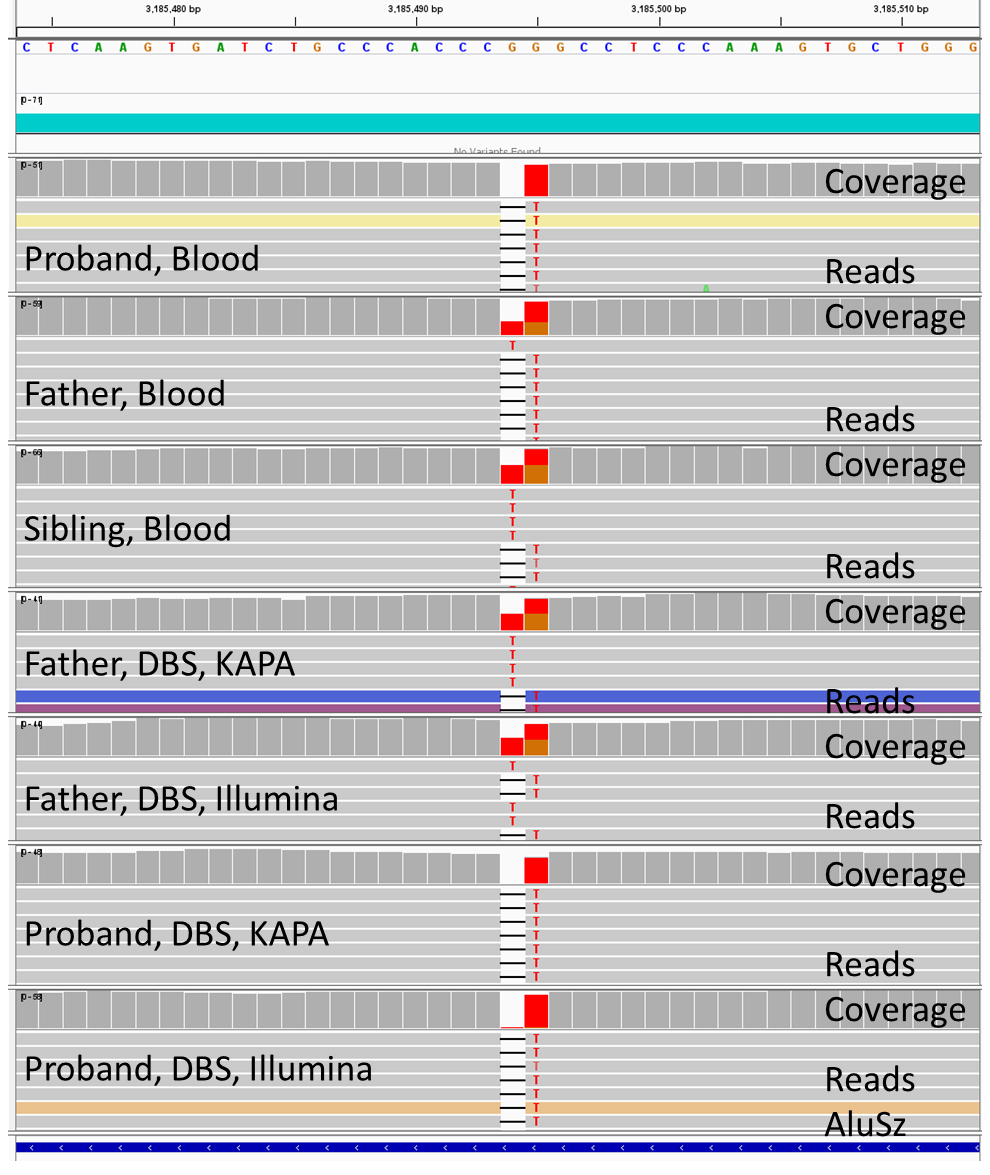

Supplement: Supplementary file 1 — Supplementary Material [file 41525_2023_349_MOESM1_ESM.docx]
