# Supplementary material for: Scalable, high quality, whole genome sequencing from archived, newborn, dried blood spots
Source: NPJ Genom Med. 2023 Feb 14;8:5. doi: 10.1038/s41525-023-00349-w (PMC9929090; doi:10.1038/s41525-023-00349-w)
Supplement: Supplementary file 2 — Reporting Summary [file 41525_2023_349_MOESM2_ESM.pdf]

nature portfolio

Corresponding author(s): Owen, M

Last updated by author(s): December 26, 2022

Reporting Summary

Nature Portfolio wishes to improve the reproducibility of the work that we publish. This form provides structure for consistency and transparency in reporting. For further information on Nature Portfolio policies, see our Editorial Policies and the Editorial Policy Checklist.

Statistics

For all statistical analyses, confirm that the following items are present in the figure legend, table legend, main text, or Methods section.

n/a	Confirmed	
☐	☒	The exact sample size (*n*) for each experimental group/condition, given as a discrete number and unit of measurement
☐	☒	A statement on whether measurements were taken from distinct samples or whether the same sample was measured repeatedly
☐	☒	The statistical test(s) used AND whether they are one- or two-sided<br>*Only common tests should be described solely by name; describe more complex techniques in the Methods section.*
☐	☒	A description of all covariates tested
☒	☐	A description of any assumptions or corrections, such as tests of normality and adjustment for multiple comparisons
☐	☒	A full description of the statistical parameters including central tendency (e.g. means) or other basic estimates (e.g. regression coefficient) AND variation (e.g. standard deviation) or associated estimates of uncertainty (e.g. confidence intervals)
☒	☐	For null hypothesis testing, the test statistic (e.g. *F*, *t*, *r*) with confidence intervals, effect sizes, degrees of freedom and *P* value noted<br>*Give P values as exact values whenever suitable.*
☒	☐	For Bayesian analysis, information on the choice of priors and Markov chain Monte Carlo settings
☒	☐	For hierarchical and complex designs, identification of the appropriate level for tests and full reporting of outcomes
☒	☐	Estimates of effect sizes (e.g. Cohen's *d*, Pearson's *r*), indicating how they were calculated

Our web collection on statistics for biologists contains articles on many of the points above.

Software and code

Policy information about availability of computer code

Data collection	DRAGEN v.4.1 is available at https://www.illumina.com/products/by-type/informatics-products/dragen-bio-it-platform.html.
Data analysis	DRAGEN v.4.1 is available at https://www.illumina.com/products/by-type/informatics-products/dragen-bio-it-platform.html. Manta v.1.6.0 is available at https://github.com/Illumina/manta. CNVnator v.0.4.1 is available at https://github.com/abyzovlab/CNVnator. vcfeval v.3.12.1 is available at https://github.com/Illumina/hap.py/blob/master/src/python/Haplo/vcfeval.py. IGV v.2.15.4 is available at https://software.broadinstitute.org/software/igv/download. Fabric Enterprise v.6.15.9 is available from Fabric Genomics Inc.

For manuscripts utilizing custom algorithms or software that are central to the research but not yet described in published literature, software must be made available to editors and reviewers. We strongly encourage code deposition in a community repository (e.g. GitHub). See the Nature Portfolio guidelines for submitting code & software for further information.

Data

Policy information about availability of data

All manuscripts must include a data availability statement. This statement should provide the following information, where applicable:

- Accession codes, unique identifiers, or web links for publicly available datasets
- A description of any restrictions on data availability
- For clinical datasets or third party data, please ensure that the statement adheres to our policy

Whole genome sequencing data are subject to conditions of the IRB protocols and CDPH policies under which the data was generated, and therefore the raw sequencing data is unavailable. For additional summary or aggregate level data, please contact Dr. Stephen Kingsmore, skingsmore@rchsd.org.

Human research participants

Policy information about studies involving human research participants and Sex and Gender in Research.

Reporting on sex and gender	Gender was not collected. De-identified blood samples from children of both sexes were utilized.
Population characteristics	DBS samples were from two cohorts: The first consisted of 25 children aged less than 18 years, of male and female sex, who received WGS from whole blood at the Rady Children's Institute for Genomic Medicine (RCIGM) between January 2018 and June 2019 for diagnosis of a suspected genetic disease. WGS was performed in laboratories accredited by the College of American Pathologists (CAP) and certified through Clinical Laboratory Improvement Amendments (CLIA). They had either been consented under various research protocols approved by the UCSD IRB and the Johns Hopkins IRB or were sample retains from clinical diagnostic testing. Blood spots were created using two paper types (filter chemical treated and non-treated) and stored for these individuals. Two cases had additional family members available for analysis: sample 6 had an affected sibling and father available, and sample 8 had father's genome available (Table 1). WGS data from DBS were compared with prior, clinical WGS data from whole blood. The second cohort were randomly selected, de-identified California Department of Public Health (CDPH) DBS samples from newborns of male and female sex without associated clinical data.
Recruitment	See population characteristics
Ethics oversight	This study received a waiver of consent from the Rady Children's Hospital and University of California – San Diego (UCSD) institutional review boards (IRB) and was undertaken as a quality improvement project.

Note that full information on the approval of the study protocol must also be provided in the manuscript.

Field-specific reporting

Please select the one below that is the best fit for your research. If you are not sure, read the appropriate sections before making your selection.

☒ Life sciences ☐ Behavioural & social sciences ☐ Ecological, evolutionary & environmental sciences

For a reference copy of the document with all sections, see nature.com/documents/nr-reporting-summary-flat.pdf

Life sciences study design

All studies must disclose on these points even when the disclosure is negative.

Sample size	Dried blood spots were generated from 92 individuals to assess quality of whole genome sequences. None of the whole genome sequences failed quality control indicating the sample size was sufficient to determine that failure occurred in approximately less than 1% of individuals examined.
Data exclusions	No data generated herein was excluded from analysis. All patient identifiers have been removed from all data to protect privacy and confidentiality.
Replication	Replication studies of the methods were undertaken and were reported herein All attempts at replication were successful.
Randomization	Dried blood spots were generated from all individuals. There was no randomization. The covariates relevant to analyses of the quality of whole genome sequences generated from dried blood spots were the age of dried blood spots., filter paper type, lysis method, library preparation method, and sequencing flowcell.
Blinding	Blinding was not relevant to this study since only objective, quantitative measurements were analyzed.

Reporting for specific materials, systems and methods

We require information from authors about some types of materials, experimental systems and methods used in many studies. Here, indicate whether each material, system or method listed is relevant to your study. If you are not sure if a list item applies to your research, read the appropriate section before selecting a response.

Materials & experimental systems

n/a	Involved in the study
☒	☐ Antibodies
☒	☐ Eukaryotic cell lines
☒	☐ Palaeontology and archaeology
☒	☐ Animals and other organisms
☒	☐ Clinical data
☒	☐ Dual use research of concern

Methods

n/a	Involved in the study
☒	☐ ChIP-seq
☒	☐ Flow cytometry
☒	☐ MRI-based neuroimaging
